# Characterization of potential driver mutations involved in human breast cancer by computational approaches

**DOI:** 10.18632/oncotarget.17225

**Published:** 2017-04-19

**Authors:** Barani Kumar Rajendran, Chu-Xia Deng

**Affiliations:** ^1^ Cancer Research Centre, Faculty of Health Sciences, University of Macau, Macau SAR, China

**Keywords:** driver mutations, breast cancer, cancer drivers, breast cancer driver genes, genetic mutations

## Abstract

Breast cancer is the second most frequently occurring form of cancer and is also the second most lethal cancer in women worldwide. A genetic mutation is one of the key factors that alter multiple cellular regulatory pathways and drive breast cancer initiation and progression yet nature of these cancer drivers remains elusive. In this article, we have reviewed various computational perspectives and algorithms for exploring breast cancer driver mutation genes. Using both frequency based and mutational exclusivity based approaches, we identified 195 driver genes and shortlisted 63 of them as candidate drivers for breast cancer using various computational approaches. Finally, we conducted network and pathway analysis to explore their functions in breast tumorigenesis including tumor initiation, progression, and metastasis.

## INTRODUCTION

Breast cancer affects women life drastically and nearly 1.7 million new cases worldwide are being identified every year since 2012 and it contributes more than 25% of the all kinds of newly identified cancer cases (http://www.cancer.org/) [1, 2]. Apart from a series of extrinsic factors promoting the occurrence, many genetic settings (intrinsic factors) drive breast cancer initiation and progression significantly. The activation of oncogenes and deactivation of tumor suppressor genes (TSGs) largely affect the maintenance and integrity of cells leading to tumorigenesis [3]. Although not all TSGs are vulnerable to mutations yet other genetic mechanisms indirectly interrupt their expressions and functions resulting in tumorigenesis [4]. In humans, several genes such as *TP53, BRCA1, BRCA2, PTEN, ATM, p27, Skp2, RAD51, etc*. are well known TSGs, which are involved in DNA repair and cellular mechanisms [5, 6]. TSGs are further classified into gatekeepers or caretakers based on their functions. Apart from tumor suppressors, a group of genes like *PUM1, B2M, ACTB, RPL13A, LDHA, NONO*, etc. are reported as housekeeping genes playing basic cellular functions (governing or preventing cell growth) and mutations in these genes promote cell proliferation [7]. In contrast, caretaker genes are mainly involved in the healthy maintenance of cells by encoding products, which stabilize the entire genome and protect genes from mutational events. Investigation of biological pathways affected by mutations of these genes will help us to understand the determinants of cancer initiation, progression, and other biological functions [8–10]. The advancement in the next generation sequencing and their allied computational techniques have paved way to identify large numbers of breast cancer gene mutations and their impacts [11]. In every cancer type, a set of significant gene mutations will strongly associate with tumorigenesis by being growth advantageous for the carcinogenic cells and those genes are known as driver genes [12]. In many breast cancer cases a significant numbers of somatic mutations as well as considerable number of germline mutations are found which are tumor enhancers and impose the risk of breast cancer tumorigenesis. Most of the driver mutations occur at somatic level, while a small number of mutations are passed on to lineages, which cause for 5 to 10% of all familial breast cancers types [13]. The most recurrently mutated published driver genes are *AKT1, GATA3, PIK3CA, MAP3K1* and *TP53* [14–17]. Apart from these genes, many other genes such as *CBFB, RUNX1* are involved in somatic mutations in breast cancer. Deletion or translocation events in tumor suppressor genes such as *AKT3* & *MAGI3* genes lead to functional abnormalities and initiates breast tumorigenesis. Recent studies on breast cancer driver genes uncovered a list of genes such as *CCND1, ERBB2, FGFR1, MYC, PIK3CA, PTEN, GATA3, MAP3K1*, and RB1 etc., which are responsible for breast cancer [18, 19].

## SIGNIFICANT DRIVER GENES ARE REAL MARKERS OF BREAST CANCER

Genetic mutations are rare and occur due to truncation, frame shift, insertions and deletions (indels), amplification and splicing abnormalities, etc. leading to loss or gain of functions. In breast cancer, over 30626 significant mutations are reported and many of them affect function of single gene or group of genes which leads to cancer progression. Its worthwhile to note that a specific genetic change cause for adverse effects i.e. neoplastic transformation. *BRCA1* and *BRCA2* genomic insertions, deletions or single nucleotide polymorphisms are also major founder mutational events and show high-risk in many breast cancer cases [20, 21]. While, a few gene mutations such as breast cancer gene 1 and 2 *(BRCA1/2*) instigate up to 25% breast cancer and also responsible for the highest number of mortalities [22, 23]. In addition to germline mutations, *BRCA1/2, PTEN, CDH1*, and *STK11* gene mutations are associated with specific disorders such as Cowden syndrome, hereditary diffuse gastric cancer syndrome and Peutz-Jeghers syndrome (https://seer.cancer.gov/archive/csr/1975_2012/) [24–26]. The mutated tumor suppressor gene, *PALB2* largely affects *BRCA2*, which increase the risk of *BRCA1/2* based breast cancer [27–30]. Apart from aforementioned genes *CDH1, STK11, PALB2, CHEK2, BRIP1, CDKN2A, CTNNB1, MLH1, MSH2, MSH6, NBN, RAD50, RAD51, TP53*, etc. are having strong association with breast cancer. Frequent gene mutations resulting in variations in single nucleotide polymorphism (SNP), copy number variations, etc. exhibit significant impacts on tumor development, these kind of genes are called driver mutation genes (http://www.cancer.org/) [8, 31]. Among the known breast cancer genes, *ATM* gene abnormality causes the development of breast cancer *RAD51C*, and *TP53* also play a strong role in the initiation and progression of breast cancer [32, 33]. The *BRIP1* gene mutations lead to high risk of both breast and ovarian cancer, whereas *MRE11A* gene abnormality is linked to ataxia-telangiectasia along with cancer [34]. Mre11, Rad50, and Nbs1 form MRN complex, which facilitate DNA repair and also reported that *NBN* gene encoding Nbs1, has the strong association with breast cancer [35]. Somatic mutations and their role in breast cancer disposition have been revealed in earlier breast cancer related studies and it is also found that genes like *ATM*, *PTEN*, etc. play major role in several germline point mutations [36–38]. Along with point mutations, insertions, and deletions, a significant number of missense mutations occur in various genes, which raise the breast cancer susceptibility [39–41]. Among the aforementioned known breast cancer driver genes, a tumor suppressor (TSG), *TP53*, is the top-mutated gene, with nearly 100% risk of breast cancer [42, 43]. A germline mutation of *TP53* also causes Li-Fraumili and Li Fraumeni-like syndromes that claim more than 40% of familial cancer [44, 45]. In addition, it also causes autosomal dominant disorders characterized by predisposition of several early inceptions of cancers, many of which are conveyed with homozygous mutant genotype with cancer relapse, and high probability of progressive and secondary cancer [46–48].

## POSSIBLE DRIVER GENES MUTATIONS IN BREAST CANCER

Identification of cancer drivers is indeed the most challenging task in cancer research and many cancer drivers are predicted using several computational and statistical methods and validated with true expression levels in cancer [49]. The genetics home reference (https://ghr.nlm.nih.gov) published list of genes such as *BARD1, BRCA1, BRCA2, CASP8, CHEK2, CTLA4, CYP19A1, FGFR2, H19, LSP1, MAP3K1, MRE11A, RAD51C, STK11, TERT, TOX3, XRCC2, GATA3, PIK3CA, AKT1, CDH1, RB1, TP53, PTEN* and *XRCC3*, and suggested that these are the most susceptible genes involved in driver mutation and having a strong association with breast tumorigenesis [11]. The copy number variations (CNVs) and single nucleotide variations (SNVs) are major root of driver mutations in breast cancer [50]. Stephens et al. (2012) reported that the numbers of mutations in protein-coding genes are remarkably unique between individuals along with list of driver mutations responsible for tumorigenesis. Most frequently mutated genes in breast cancer are *TP53, ERBB2, GATA3, FGFR1, CCND1* and *PIK3CA* [51]. A number of genes which are involved in breast tumorigenesis and act as potential drivers are *ARID5B, CDH1, CTCF, HDAC9, KDM5B, NCOR2, SETD1A, SXL2*, etc. Some of these genes encode proteins that control chromatin structure whereas other driver genes, such as *ATR*, and *FANCA* are mainly involved in DNA repair pathway. The recent whole genome sequencing (WGS) of 560 breast cancers samples identified 89 genes and 2433 breast cancer sequencing projects identified 40 breast cancer drivers genes from ER positive and ER negative breast cancer subtypes and many of these genes such as *ARID1A, CTNND1, NUP107, CHD8, FANCI, CHD9, CTCF, KEAP1, PCDH18, LAMA2, HDAC9, ARFGEF1, MLLT4, FOXO3, CDKN2A, MAP3K1, GPS2, CTCF, CDH1, GATA3, AKT1*, etc. have diversified functional change mutations [17, 52]. However, each published data on cancer drivers reported a distinct set of driver genes with few overlaps and so far, no standard approach is developed to identify and validate breast cancer driver genes [53].

## CURRENT TRENDS IN SCREENING OF GENES INVOLVED IN BREAST CANCER DRIVER MUTATIONS

Driver gene mutations are necessary tool for the characterization of cancer phenotype, since they mainly affect gene expression followed by miscoding of amino acids, which provoke functional changes at protein level. However, passenger genes replicate many folds during DNA replication events without any extricating functional impacts [54, 55]. Voluminous methodologies have been employed to predict and identify breast cancer driver mutation genes, including computational identification, statistical testing, and, so on. Genetic mutational screening is one of the most widely used methods for the identification of mutations in germ cells based on looking at the family history of breast cancer [56, 57]. Driver mutation frequency is largely interrelated with breast cancer subtypes for example, *TP53* mutation frequency is many folds higher in basal-like than other breast cancer subtypes [11]. Statistical analysis yield better results in driver genes identification, it also predicts high-frequency cancer driver genes using oncogenic tree model construction [58]. Thus, breast cancer driver prediction methodologies depend on key factors such as, the number of samples used for analysis, mutational patterns, frequency and function modifying mutations, etc. Several efficient tools exist to predict the mutation drivers, though each tool works using its own hypothesis/algorithms with diverse limitations. Accordingly, each driver mutation recognition protocol delivers distinctive results from one another. In this paper, we used several intensive computational driver gene identification approaches, tools, resources, etc. for the identification of most impressive driver mutation genes and their role in breast tumorigenesis.

## COMPUTATIONAL APPROACHES FOR DISTINGUISHING BREAST CANCER DRIVER GENES MUTATION

Predicting breast cancer driver gene is a cumbersome task, as it generates a lot of false positive data and corroborating those results are most challenging. In this study, we used a dozen of computational driver gene identification approaches including online resources, offline and online tools to explore most potential breast cancer driver genes to avoid limitations of each approach. These include, the cBioportal (www:cbioportal.org/), The Cancer Genome Atlas (TCGA), International Cancer Genome Consortium (ICGC), 1000 Genomes, Catalogue of Somatic Mutations in Cancer (COSMIC), Human Cancer Database (http://db.cngb.org/cancer/), National Cancer Database (https://www.facs.org/quality-programs/cancer/ncdb), OASIS (http://oasis-genomics.org/) and many other useful cancer resources (Table 1). Apart from above-mentioned resources, Pan-Cancer (https://www.synapse.org/) developed by TCGA database is very efficient resource which provides analyzed cancer data, including mutation profiles, copy number variations, gene expression information, microRNA, etc. [59–62]. Included in Table 1 also several other methods that have been recently designed to find potential breast cancer driver genes by computational and statistical techniques, such as IntOGen, Driver DBV2, MutSigCV, etc.

**Table 1:** List of driver identification methods used to incorporates the prediction of breast cancer driver genes, their working principle and supporting references

Driver Identification Method	Driver Gene Identification Principle	Citations
IntOGen	Identifies alterations at transcriptomics level, CN gain and losses in tumor sample. It also integrates OncodriveFM for the identification of accumulation mutations, background mutation rate and OncodriveCLUST for mutation cluster identifications. Further, SIFT, Polyphen and Mutation Assessor are used to predict the impact of mutations.	[62–65]
SIFT	Amino acids substitutions and their deleterious impacts prediction. It find the homologous sequences using PSI-BLAST followed by picking sequences with specific diversity and calculating the SIFT scores.	[66]
PolyPhen-2	Analyzes non-synonymous SNP using multiple sequence alignment and structure information followed by predicting the probabilistic damaging variants with confidence prediction and at last interpret the results with mutational impact.	[67]
Mutation Assessor	Predicts mutational impact by calculating functional impact score derived from addition of conservation score and specificity score.	[68]
Driver DBv2	Uses large exome and RNAseq datasets to predict the driver genes using several incorporated tools.	[69]
Active Driver	It identifies significant mutations of cancer genes in active sites of proteins such as mutations in signaling proteins or domains or regulatory elements. It uses gene-centric logistic regression model including multiple factors to estimate mutation significance.	[70]
Dendrix	This algorithm discovers driver genes with high coverage and high specificity using mutation data.	[71, 72]
MDPFinder	It combines mutation and expression data to validate the driver genes and their mutated pathways.	[73]
Simon	It identifies functional mutation impact on proteins, variations in background mutation frequency and genetic code redundancy among tumors.	[74]
NetBox	It identifies the driver genes by comparing genes and performing network analysis on human interaction Network (HIN) data.	[75]
MutSigCV	It uses overall mutation rates and distribution patterns and analyzes background mutation rates with patient specific as well as gene specific mutation rates. Finally it includes expression levels and replication periods.	[76]
MEMo	It identifies the driver genes based on recurrently mutated genes among tumor data with consistent mutational specificity.	[77]
e-Driver	It manipulates internal distribution of somatic functional missense mutations amongst functional domains by relating mutation rates with other regions of same protein.	[78]
DawnRank	Uses gene expression data to construct gene network and rank them based on impact and it analyzes somatic alteration data to identify personalized driver alterations.	[79]
DriverNet	Driver genes are identified based on genomic aberration states of various patients, genes, gene expression data and it further takes biological pathway data into account and builds the network driver genes.	[80]
MSEA	It predicts cancer driver genes based on patterns of mutation hotspot.	[81]
iPAC	Identifies non-random somatic mutations in protein using tertiary protein structure information.	[82]
CoMDP	It uses mutation data to identify driver genes and their pathways. It also predicts genes with other multiple co-occurring biologically significant pathways.	[83]

Mutation modeling and evaluating number of deleterious mutations in breast cancer are also employed to predict potential driver genes and massive statistical testing is carried out to predict the prompt driver genes and their functional domains [84]. Computational modeling, gene pathway and network analysis are other feasible techniques proposed to identify most probable driver genes [49]. DrGaP is a tool that predicts driver genes and their signaling pathway using statistical analysis [76]. Apart from aforesaid tools, many viable techniques, tools, and databases provide significant driver gene mutations, and mutational significance of genes involved in single/multiple cellular pathways, etc. The OASIS web portal is also one of comprehensive resource providing tons of information about somatic mutation, gene expression, copy number alteration, etc. from normal, tumor cases, and cell lines (http://oasis-genomics.org/) [77]. This web portal, fetch primary genetic and metabolic pathway analyses data from Pan-Cancer project, COSMIC, BIOCARTA (http://www.biocarta.com), KEGG (http://www.genome.jp/kegg/pathway.html), etc. [78].

Apart from abovementioned resources for driver gene identification, we have also validated the genetic interactions through various approaches. To test the capability of identifying the driver genes in genetic interaction level, we constructed FunCoup (functional Coupling) database to explore the functional relationship between genes and their functions [79]. Genetic network is most significant method to derive genetic as well as functionally associated genes using Genemania web server and it predicts gene functions by integrating several functionally associated networks [81]. The consequent level of network analysis is performed using MUFFINN (MUtations For Functional Impact on Network Neighbors). MUFFINN is one of the efficient programs for identifying most common driver genes by mutation frequency and most linked pathway neighbors [82, 83]. MUFFINN uses a pathway-centric approach and it also identifies the top 1000, 500, 100 interacting gene clusters along with network constructed using HumanNet and String (Search Tool for the Retrieval of Interacting Genes/Proteins) Database [85, 86]. To further validate the identified driver genes and their genetic interaction network construction we used FunRich (Functional Enrichment) program. FunRich analyzes genes and their interacting partners based on comprehensive information obtained from various renowned databases with strong annotations [87]. SIFT algorithm is used for functional impact and validation of identified driver genes. SIFT is one of the most powerful algorithms used to identify and evaluate detrimental effects of genetic variations in driver genes and their impacts at protein level. PolyPhen2 is another potential tool, which predicts the probabilities of amino acid substitutions and its collective impacts on structural and functional tendency [88, 89]. Thus, every driver gene prediction approach has some representative strength to identify the real cancer driver genes and this ends with the major concerns. For example, a frequency-based approach always needs large number of samples to possibly identify the rarely mutated cancer drivers [90].

**Figure 1:**
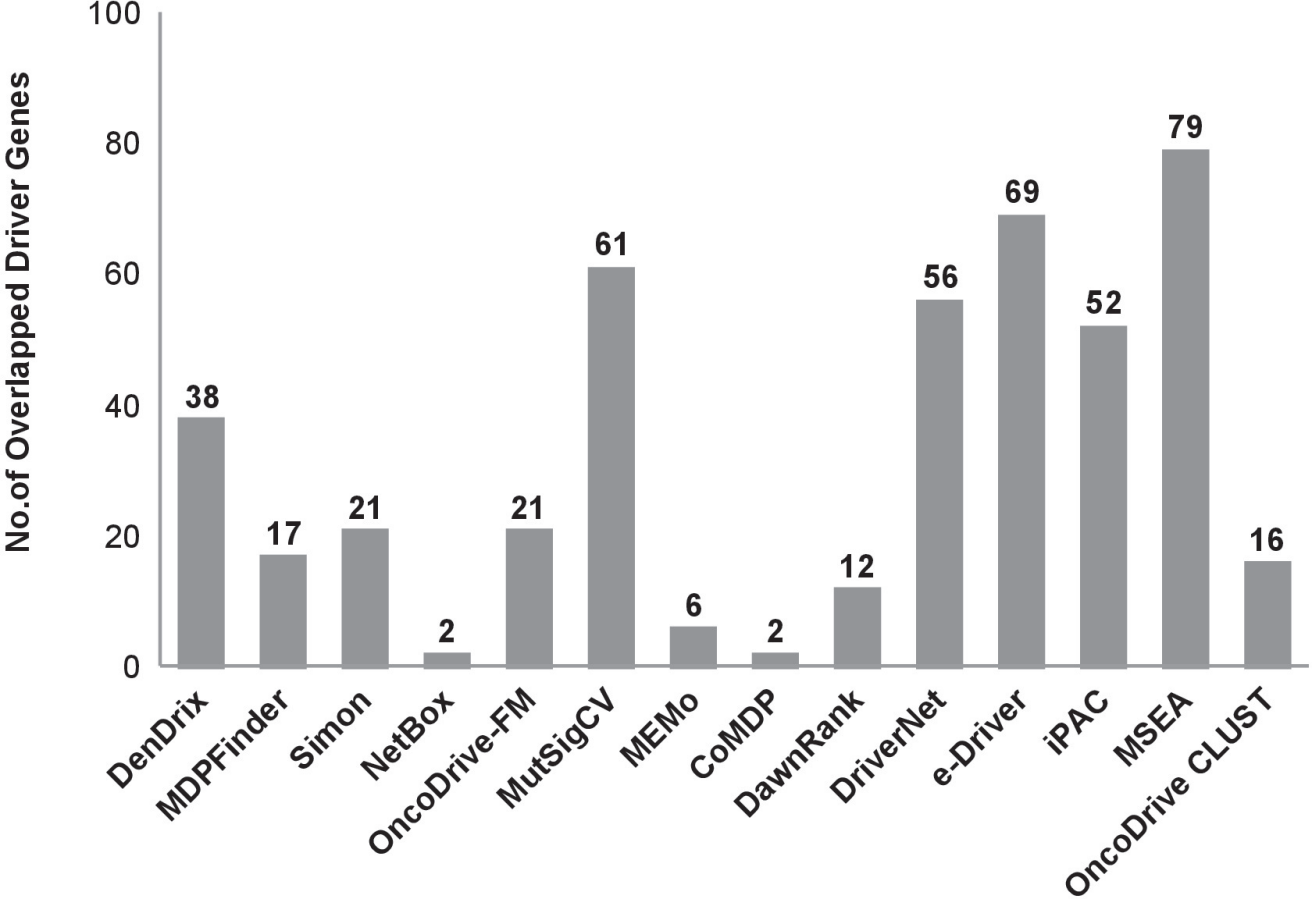
Total number of breast cancer driver genes identified using various computational methods

Nevertheless, through this study, we established that driver gene identification is purely based on mutations in key genes, which are really driven, by functional mutations. Every Driver DB associated algorithms are working in a distinctive way and it yields various outputs. For example, algorithms such as Active Driver focus on phosphorylation and kinase domain site. Similarly Dendrix, MDP Finder, Oncodrive-FM and MutSigCV predict based on mutational specificity, high impact mutational accumulation and patient-specific mutations respectively. Hence, Driver DB associated tools provide comprehensive ways of predicting drivers based on several criteria such as recurring mutations, accumulation of mutation with high functional impact, mutual exclusivity and the spectrum of mutation, gene expression data, background mutation rate, etc. resulting in 956 breast cancer drivers identified from various breast cancer subtypes (detailed list of driver genes is given in Supplementary Table 1). The driver genes are filtered out and further shortlisted based on more than one Driver DB associated tools, which report the genes with hotspots of mutation, missense mutation, etc. From the initial filtering 452 genes were obtained and further redundant genes were removed leaving 195 driver genes, which are chosen for further analysis (Figure 1). The ICGC database (https://icgc.org/icgc) is used to fetch the detailed mutations data including chromosomal location, type of mutation, codon alterations, and amino acid variations and cancer subtypes of identified driver genes are retrieved, analyzed and tabulated in Supplementary Table 2. Similarly, IntOGen integrate results with various mutation-calling protocol such as OncodriveFM & OncodriveCLUST, and it identifies genes responsible for functional mutations and mutational impacts at protein level.

**Figure 2:**
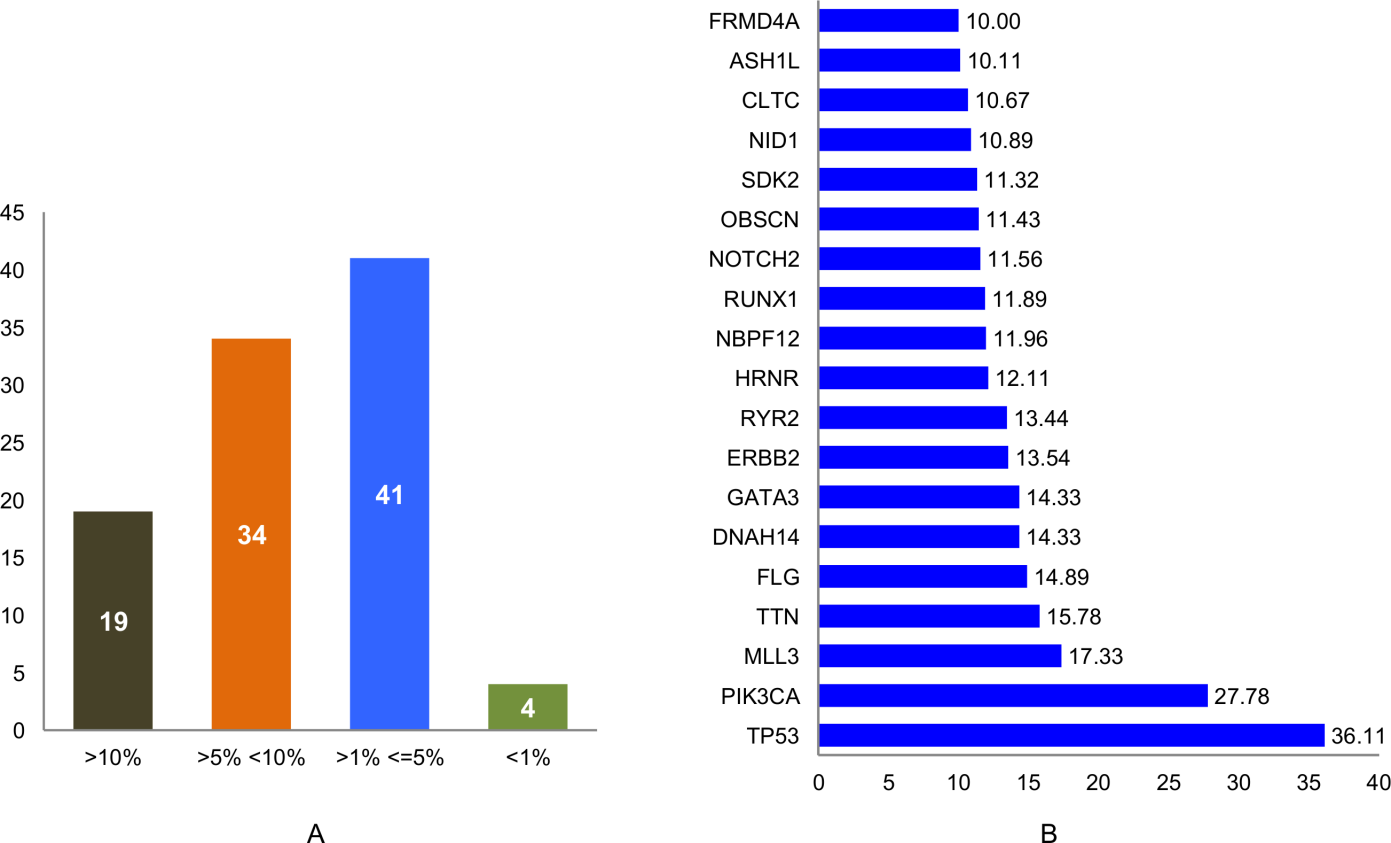
**(A)** Identified driver genes classified based on their Mutation percentage; **(B)** High percentage of mutations (>10%) are observed in the identified 63 breast cancer driver genes through the analysis of 9 breast cancer patients data analysis using cBioPortal.

**Figure 3:**
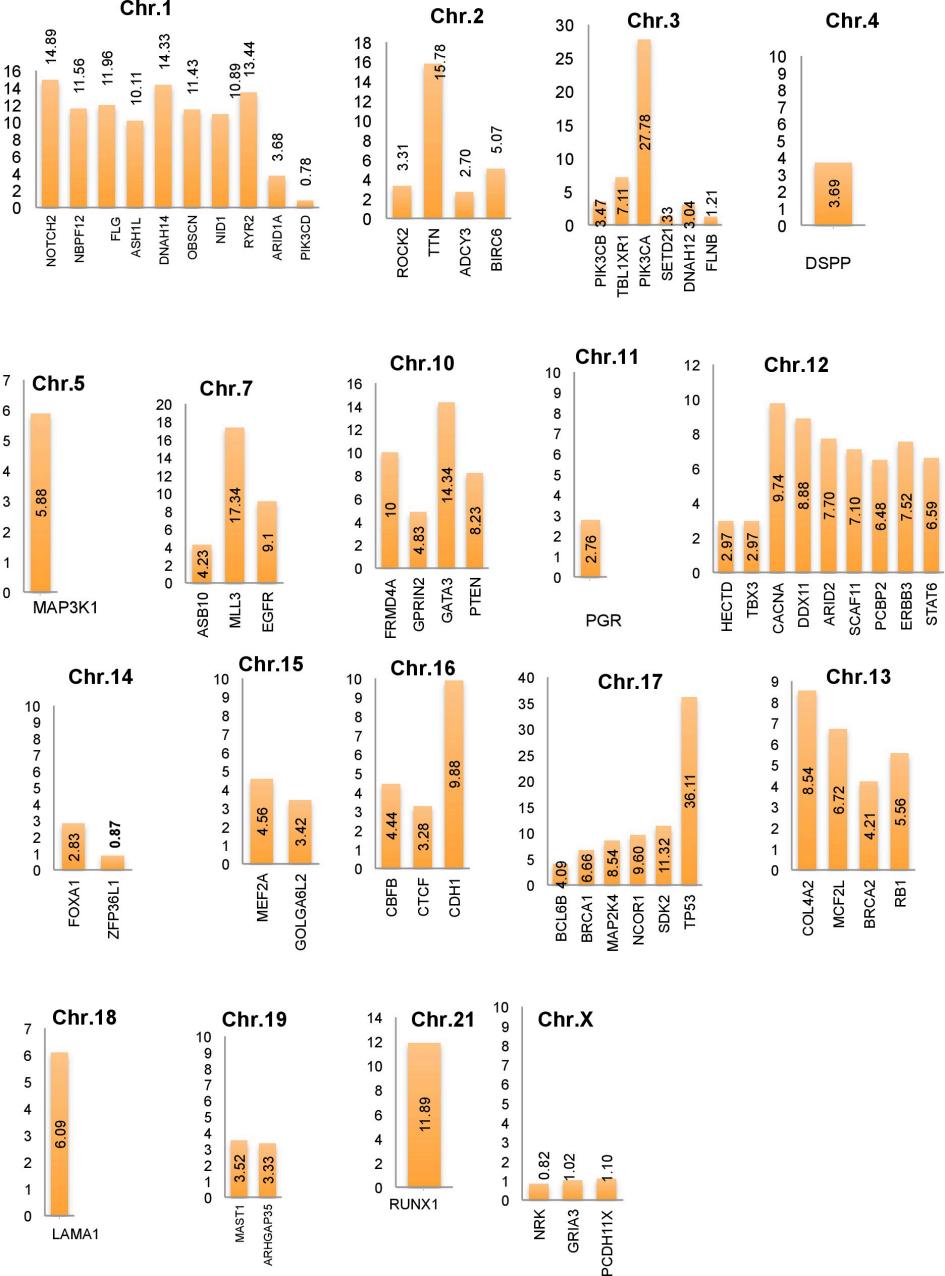
Average breast cancer gene mutations identified using cBioPortal projects (4162 breast cancer samples) along with identified top candidate driver genes and their respective chromosomes locations

In this study, a list of top candidate genes were identified through our approaches, by incorporating a selective list of efficient driver gene prediction tools and resources which were proved earlier with other type of cancer gene prediction. We used the TCGA and ICGC breast cancer data to identify frequency and type of mutations, and we found a number of new genes such as *FLG, DNAH14, NBPF12, RYR2, ARHGAP35, OBSCN, CLTC*, etc. are highly mutated in breast cancer, along with some well-known driver genes, like *TP53, PIK3CA, MLL3, PTEN, GATA3, ARID1A*. We further categorized the identified breast cancer driver genes into four major types based on the mutation percentage of each driver used for this study (Figure 2A). The analysis further extended to find the mutations frequency of breast cancer genes among the nine BRCA projects available in cBioPortal (www.cbioportal.org). We identified driver genes (*TP53* gene 36.11% followed by *PIK3CA* 27.78%, *MLL3* 15.78%, *TTN, FLG, DNAH14, GATA3, ERBB2, RYR2, HRNR, NBPF12, RUNX1, NOTCH2, OBSCN*) and overall average mutation rate is calculated and given in Figure 2B. Investigation is further continued to validate candidate driver genes and their mutation profiles in breast invasive carcinoma samples obtained from TCGA-Pan-Cancer data resource (https://www.synapse.org). Genes were chosen for further analysis based on the most frequent functional mutations such as splice site mutations, missense mutations, frame shift insertion and deletions mutations, In-frame insertion and deletions, etc. Result of this analysis identified 61,466 functionally significant gene mutations and all genes are further screened for most potential driver genes discovery. Moreover, the analysis extended to confirm the identified drivers genes and their mutational impact at functional level by using PolyPhen-2 programs for predicting the high-impact deleterious mutations along with high IntOGen variant impact score. To validate the most probable breast cancer driver genes, the results of IntOGen prediction, COSMIC, CBioPortal breast cancer data, OASIS data portal were used. As a result of intensive filtering and analysis, 63 driver genes were short-listed. The TCGA breast cancer projects were used to calculate the average mutations of individual high-confidence driver genes by chromosome-wise (Figure 3) and individual project wise (Supplementary Figure 1). Nevertheless, a significant percentage and type of mutations diversity (Missense, truncation, amplification, etc.) are found among the breast cancer projects, due to heterogeneity, individual gene mutations, and patient specific clinical factors, etc.

## FUNCTIONAL EFFECTS OF BREAST CANCER DRIVERS IN TUMORIGENESIS

Several driver gene prediction tools exist to evaluate potential driver genes based on their functional mutations and impact and also use transcriptomics data to reveal potential driver genes at the protein level [91]. Although numerous computational techniques identify and classify the driver genes based on the mutations and functional impact, yet *in vitro* and *in vivo* assays are necessary for further validation. In breast cancer, many known genes are considered to be an effective driver genes including *BRCA1/2, TP53, PIK3CA, GATA3*, etc., which govern the most cancer pathways. Besides identified and known driver genes, many novel genetic elements are actively involved in breast cancer metabolic pathway. In this study, we have identified and propose numerous novel breast cancer driver genes, which are validated using various computational techniques. From the list of identified driver genes, titin (*TTN*) gene is one of the important genes with an average of 15.78% mutation rate in breast cancer while earlier studies also revealed that *TTN* is highly mutated in other cancers [92, 93]. Similarly, filaggrin (*FLG*) gene is a highly mutated driver gene, which had an average mutation of 14.89%. *FLG* gene mutations are found in several other cancer types such as non-melanoma skin cancer, head and neck cancer, lung cancer, colorectal cancer, uterine cancer, prostate cancer, etc. [94]. Hence, the *FLG* gene may also have a strong association with multiple carcinomas. The obscurin (*OBSCN*) gene is identified as one of most frequent driver genes in all our analysis, and an average mutation rate is 11.44%. *OBSCN* gene is vastly mutated in various cancer types and this gene mutation leads to giant obscurins protein loss followed by high susceptibility of breast epithelial cells to DNA damaging elements [92, 95]. Earlier studies revealed that *OBSCN* gene stimulated survival of breast epithelial cell and prevented cell apoptosis [96]. Hence, *OBSCN* gene is one of the potential breast cancer drivers and also has a strong association with other cancer types.

In addition to aforementioned genes, few driver genes may act as tumor suppressor, oncogenes, gatekeepers, and caretakers, etc. AT-Rich Interaction Domain 2 (*ARID2*) as a tumor suppressor gene is frequently mutated driver gene identified in all our analysis (Mutation average is 7.7%). ARID2, as a variant gene of SWI/SNF complex, mutation has strong associations with huge number of cancers especially in hepatocellular carcinoma, gastric cancers and breast cancer [97–99]. Rho-Associated Coiled-Coil Containing Protein Kinase 2 (ROCK2) is another important driver gene identified in this analysis, although its overall mutation frequencies are comparatively low (3.32%). Previous research on ROCK2 gene and its relevance to breast cancer are proven and a critical amino acid mutation (T431N) is identified as the high-risk factor in breast cancer metastasis. In addition to the above-mentioned functions of identified high-confidence driver genes (excluding published driver genes), we performed an intensive literature search to corroborate and strengthen our approach. The data including genes involved in various cancers, functions, and pathways along with supporting citations are tabulated (Table 2).

**Table 2:** Identified top candidate breast cancer driver genes (other than known driver genes) and their functional backgrounds

Identified Driver Genes	Cancer Type	Related pathway	Known Functions	References
ADCY3	Gastric cancer	cAMP/PKA/CREB pathway	Increased cell migration, invasion, and proliferation, which are characteristic of cancer.	[100–102]
ARHGAP35	Osteosarcoma, Breast cancer, Pancreatic carcinoma	Regulation of RhoA activity and focal adhesion and migration	Human glucocorticoid receptor DNA binding factor	[103–105]
ARID2	Hepatocellular carcinoma/melanoma	Chromatin Remodeling	Activating ligand dependent transcription by nuclear receptor	[98, 105]
ASB10	Glioblastoma multiform, Ovarian Cancer	Cytokine signaling	Ubiquitination and Ubiquitin protein ligase binding	[106]
ASH1L	Liver cancer;Leukemia; breast cancer	Tight junction and lysine degradation	Chromatin regulator; Site specific lysine methylation on histone and other proteins	[107–109]
BCL6B	Breast cancer;Gastric cancer	P53, MAPK and cancer related pathways	Nucleic acid bindingTumor suppressor gene in gastric cancer	[110, 111]
BIRC6	Breast cancer;	Apoptosis and Autophagy	miRNA dependent apoptosis induction	[112]
CACNA1C	Breast cancer, Gastric, colorectal, pancreatic, leukemia, brain, skin, prostate cancer	Circadian entrainment and NFAT and Cardiac Hypertrophy	High alteration in Ca^2+^ ion it accelerates cell proliferation, migration and up-regulation in breast cancer	[113–115]
COL4A2	Cardiovascular disease and intracerebral hemorrhage, glaucoma, etc.	Interleukin-3, 5 and GM-CSF signaling and Pathways in cancer.	Regulation of angiogenesis and tumor growth	[116, 117]
DDX11	Breast cancer, Fanconi Anemia	Golgi and subsequent modification and unfolded protein response	Genome stability	[118]
DNAH12	Prostate cancer	Respiratory electron transport, ATP synthesis chemiosmotic coupling and uncoupling protein for heat production	ATP binding andRegulatory function	[119]
DNAH14	Ovarian cancer	Respiratory electron transport, ATP synthesis chemiosmotic coupling and uncoupling protein for heat production	ATP binding andRegulatory function	[120]
DSPP	Oral squamous cell carcinomas;Prostate and breast cancer	ECM proteoglycan and degradation of the extracellular matrix organization	Vital factor in dentinogenesis;	[121, 122]
FLG	Nonmelanoma cancer, head and neck, colorectal, breast, ovarian, prostate cancer	AhR pathways	Calcium ion binding	[94]
FLNB	Breast Cancer; Ovarian cancer; Colorectal cancer	MMP-9 and ERK pathway	RAS induced tumor growth	[123, 124]
FRMD4A	Gastric cancer;Rectal cancer	-	Protein Binding	[125, 126]
GOLGA6L2	Breast cancer;Hapatocellular carcinoma	-	Protein coding	[127]
GPRIN2	Rett Syndrome;Breast Cancer	-	Neurite outgrowth	[79, 128]
GRIA3	Pancreatic Cancer; Breast cancer	glutamate receptor signaling pathway	excitatory synaptic transmission	[129, 130]
HECTD4	Esophageal, non-small-cell lung and head and neck cancer	Protein modification and Ubiquitination	Ubiquitin-protein transferase activity	[131]
LAMA1	Breast cancer; Colon cancer	Cancer and Integrin pathway	Receptor binding	[56, 132]
MAST1	Breast Cancer	-	Ion/ATP/protein binding	[133]
MCF2L	Breast cancer	Rho/Rac signaling and p75 NTR-receptor-mediated signaling pathways	Rho-guanyl-nucleotide exchange factor activity	[134]
MEF2A	Breast cancer	P38 MAPK signaling	Neuronal differentiation and survival	[135, 136]
NBPF12	Neuroblastoma; small cell lung cancer neurogenetic diseases	-	CHEA Transcription factor binding site	[137, 138]
NID1	Gastrointestinal cancer	Non-integrin membrane-ECM interactions and Degradation of the extracellular matrix	Act as cross-linker with other extracellular matrix	[139–141]
NRK	Breast cancer	TNF-alpha-induced signaling pathway	Receptor signaling protein serine/threonine kinase activity and ATP binding	[142]
OBSCN	Highly mutated in various cancers including breast cancer	RhoA signaling	Structural and regulatory functions	[95, 96, 143]
PCBP2	Hepatocellular cancer; Familial breast cancer; lymphocytic leukemia, colorectal cancer	RIG-I/MDA5 mediated induction of IFN-alpha/beta pathway and mRNA splicing pathways	Transcriptional role	[144]
PCDH11X	Esophageal carcinoma, breast cancer, Prostate cancer	-	Cell adhesion	[145, 146]
PGR	Breast and Ovarian cancer	oestrogen-mediated pathways	Tumor repressing mechanism	[147, 148]
PIK3CB	Oral-squamous cell carcinoma, breast cancer and other wide range of cancer	Involved in AKT, PTEN and PIK3CA pathways	Cell cycle growth regulation	[149]
PIK3CD	Breast, Ovarian and colon cancers	PIK signaling	Transcription binding factor	[150–152]
ROCK2	Breast, lung, ovarian, intestinal cancer	RhoA signaling	Actin cytoskeleton organization, Adhesion, migration, Proliferation and apoptosis.	[153–155]
RYR2	Breast Cancer, Lung Cancer, Bladder cancer	cAMP-dependent PKA activation	Calcium ion binding, Calcium/calmodulin binding	[156, 157]
SCAF11	Lung adenocarcinoma, various cancers	Apoptosis	Protein/zinc ion/poly(A)RNA binding	[158]
SDK2	Non-small cell lung cancer	-	Adhesion, Promotes synaptic connectivity	[159–161]
STAT6	Breast cancer, Lung cancer	Integrin, Interleukin-3,5 and GM-CSF signaling pathway	IL-4 mediate cell growth regulator, inhibitIL-4 induced cell death	[125, 182, 183]
TTN	Colorectal, testis, gastric, breast, ovarian, renal cancers	Platelet activation, Signaling and aggregation pathway	Chromosome condensation and segregation	[54, 76]

Through the OASIS web portal, METABRIC, and BRCA-TCGA data were used for the identification and analysis of mutation profiles of 63 top driver genes. The details of mutational profiles with gene classifications of 63 top candidate breast cancer genes are given in Table 3. Among these drivers, we found 13 tumor suppressor genes (TSGs), ten oncogenes (OG), six gatekeepers and one gene had both OG and TSG features. More copy number loss mutations are observed in (>7%) genes such as *CDH1, CBFB, CTCF, BCL6B, MAP2K4, TP53, NCOR1, PGR, RB1*, and *BRCA2* genes. The large proportion of driver mutations occurred in protein coding exonic as well as in intergenic regions and these regions are considered as most significant genetic fragments and the actual insights of those regions are functionally significant. Subsequently most of the intergenic nucleotide bases are the regulator of adjacent genes and still many intergenic regions and functional roles remain uncertain. Thus, intergenic regions might be responsible for genetic variations that cause tumorigenesis and further insight on those intergenic regions of sequences may enlighten driver genes transforming mutations with a good understanding of tumorigenesis process [162]. In breast cancer, driver mutations also emphasize the functional impact at the protein level. Many somatic driver mutations observed in breast cancers are tumor dependent and may vary from tumor to tumor. In order to increase the reliability of prediction, six different approaches such as Cancer Genome Consortium prediction, MuSiC, OncodriveFM, OncodriveCLUST, Active Driver, MutSig were used to ensure the confidence level of potential breast cancer drivers.

**Table 3:** Mutation profiles of identified top candidate BRCA driver genes

BRCA Drivers	Substitution %	InDel %	Amplification %	Copy Gain %	Copy Loss %	Deletion (%)	Expression Outliers High %
CDH1*	3.2	0.1	-	1.4	16.5	2.9	-
**CBFB^**	1.7	-	-	1	16.4	1.3	-
CTCF	1.9	-	0.1	1.1	16	2.9	
MAP2K4*	2.4	0.4	0.2	0.4	12.2	2.5	-
BCL6B	0.1	-	0.1	0.4	12.2	1.4	-
TP53*	6.8	0.4	-	0.6	12	1.5	-
**NCOR1**+	3	0.3	0.2	0.8	11.3	1.5	-
PGR	0.5	-	0.2	2	10.8	2.8	51.4
RB1*	1.3	-	-	0.8	9.3	1.8	-
BRCA2*	1.3	-	0.2	1.8	7.4	1.2	66.5
MCF2L	0.5	-	0.4	4	6.7	2.5	-
COL4A2	0.5	-	0.3	4.1	6.5	2	-
GOLGA6L2	0.1	-	0.3	2.2	6.2	1.7	-
ARID1A*	1.4	0.1	0.1	0.3	5.6	0.8	-
SETD2*	1	-	-	0.8	5.1	0.5	-
PIK3CD	0.4	-	0.2	0.8	5.1	1.2	1.2
DNAH12	1	0.1	0.1	0.6	4.9	0.5	15.8
FLNB	1.2	0.3	0.1	0.8	4.8	0.6	-
ZFP36L1	0.5	0.1	0.1	1.4	4.7	1	-
LAMA1	1	-	0.1	2.7	4.3	1	-
BRCA1*	1	-	0.2	4.7	4.2	0.1	52.2
MAP3K1	3.5	0.1	-	2.2	4.1	0.8	-
PTEN*	1.7	0.4	-	0.7	3.9	0.9	-
ASB10	0.3	-	0.2	3.9	2.7	0.8	-
**NRK**+	0.9	-	0.1	1	2.5	0.3	-
DSPP	1.2	0.3	0.2	1.1	2.3	0.4	-
MEF2A	0.1	-	1.2	3.8	2.3	0.3	-
PCDH11X	0.8	-		0.9	2.2	0.3	-
CACNA1C	1.3	-	0.9	5.1	2.1	0.3	-
GRIA3	1.2	0.1	0.1	1.1	2.1	0.2	-
TTN	13.7	-	0.1	2	2	-	-
FOXA1	1.3	-	0.8	4.7	2	0.4	84.2
TBX3	0.9	-	-	1.6	1.9	0.2	-
**NOTCH2**^	0.8	-	0.8	4	1.8	0.2	-
**ARHGAP35**^	0.8	-	0.1	1.9	1.8	0.1	-
HECTD4	1.2	-	0.1	1.6	1.5	0.2	-
MAST1	1	-	0.2	3.4	1.4	0.1	22
**RUNX1**^	1.2	0.1	0.2	3.5	1.4	0.1	-
**ADCY3**+	0.3	-	0.1	1.9	1.4	0.1	-
ROCK2	0.5	-	0.3	1.9	1.2	0.3	-
**PCBP2**+	0.3	-	0.1	2.2	1.1	0.1	-
ARID2*	0.5	-	0.2	2.4	1	0.3	-
SDK2	1	-	1.4	11.6	1	0.4	-
OBSCN*	1.7	-	1.4	32.9	1	0.1	-
SCAF11	0.4	-	0.2	2.4	1	0.2	-
STAT6	0.1	-	0.2	2.3	0.9	-	-
GATA3*	0.9	2.4	0.9	7.3	0.8	0.2	81.1
NID1	1	0.1	1.6	33.3	0.8	0.2	-
**ERBB3^**	1.3	-	0.1	2.4	0.8	0.1	-
**DDX11+**	0.4	-	0.8	4.4	0.8	0.3	-
FRMD4A*	0.5	-	0.8	6.5	0.8	0.1	-
PIK3CB	0.6	-	0.3	4.8	0.8	-	-
BIRC6	1.3	-	0.1	2.4	0.7	0.1	-
**EGFR^**	0.6	-	0.8	4.5	0.7	0.3	-
RYR2	3.9	-	3.5	33.1	0.6	0.3	5.9
DNAH14	0.5	0.1	1.3	33.7	0.4	0.1	79.8
TBL1XR1	0.5	-	0.7	8.2	0.4	-	-
**PIK3CA^**	32.1	0.6	0.9	8	0.3	-	0.1
GPRIN2	0.1	0.3	6.4	2.9	0.3	-	-
NBPF12	0.3	-	4.5	45	0.2	0.1	-
ASH1L	1	0.1	1.4	31.2	0.1	-	-
MLL3*	0.8	-	0.8	4	1.8	0.2	-
FLG	4.4	-	2	30.9	-	0.1	-

Identified driver genes are categorized with Tumor suppressor (* with bold caption); Oncogene (^ with bold caption); Gatekeeper (+ with bold caption).

## GENE INTERACTION NETWORK ANALYSIS: IDENTIFYING DIRECT AND INDIRECT INTERACTING PARTNERS OF BREAST CANCER DRIVERS

**Figure 4:**
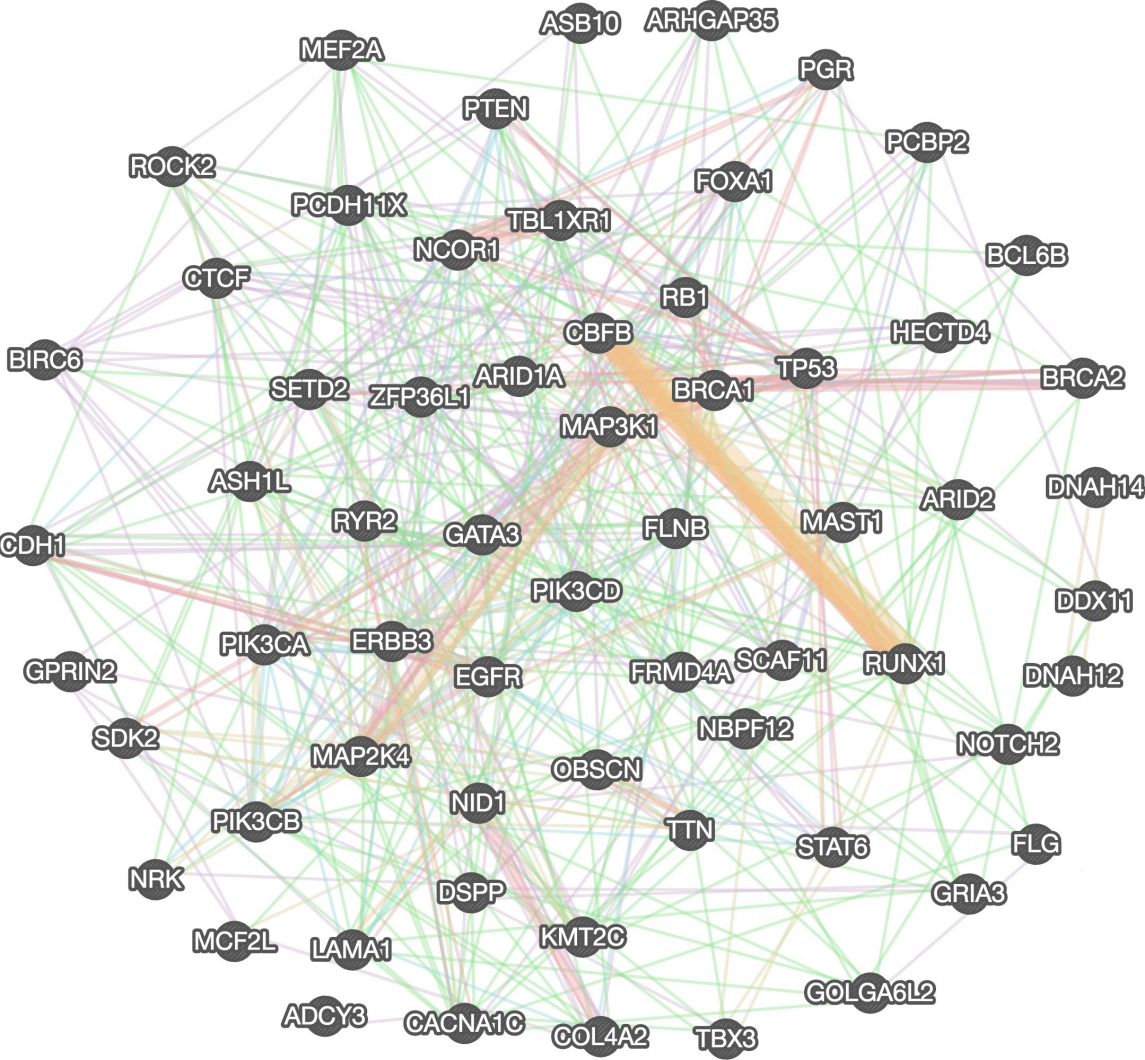
Genetic interaction network of identified top candidate breast cancer driver genes

The genetic network analysis is performed to explore more direct and indirect partners of breast cancer driver genes using FunCoup (Functional Coupling) package [85]. The FunCoup analysis is used to construct focused gene networks for driver genes and indirect genetic partners for further validation of hub (driver) genes. We constructed gene network using Genemania web server and found genetically as well as functionally associated genes among the identified driver genes to illustrate the close relationship among selected driver genes. Identified driver genes and their interaction network is constructed in Genemania server by combining several interaction network groups (n=572) obtained from various studies such as co-expression (180), co-localization (10), genetic (199), pathway related (43), physical (75), predicted interactions (9), and genes sharing protein domains (56), eventually these combined information provide more insight on molecular, functional and pathway level interaction among genes and it sorted all network groups based on Genemania score, false discovery rate (FDR) for further construction and validation of gene network (Figure 4) [85, 86]. The subsequent network analysis is further extended to cross-validate resulting driver genes using two more methods, MUFFINN and FUNRICH, which are commonly used methods to identify common driver genes by mutation frequency and most linked pathway neighbors in functional networks [66, 67]. We used top 100 neighbor genes from golden-standard databases used in MUFFINN and their mutation occurrences, and refine them for further network construction. The 63 driver genes are consistently identified through all approaches. Thus the data further confirm that these genes are most commonly mutated genes and their most damaging missense mutations flaunting highly deleterious functional impacts.

## DISCUSSION AND FUTURE ASPECTS

**Figure 5:**
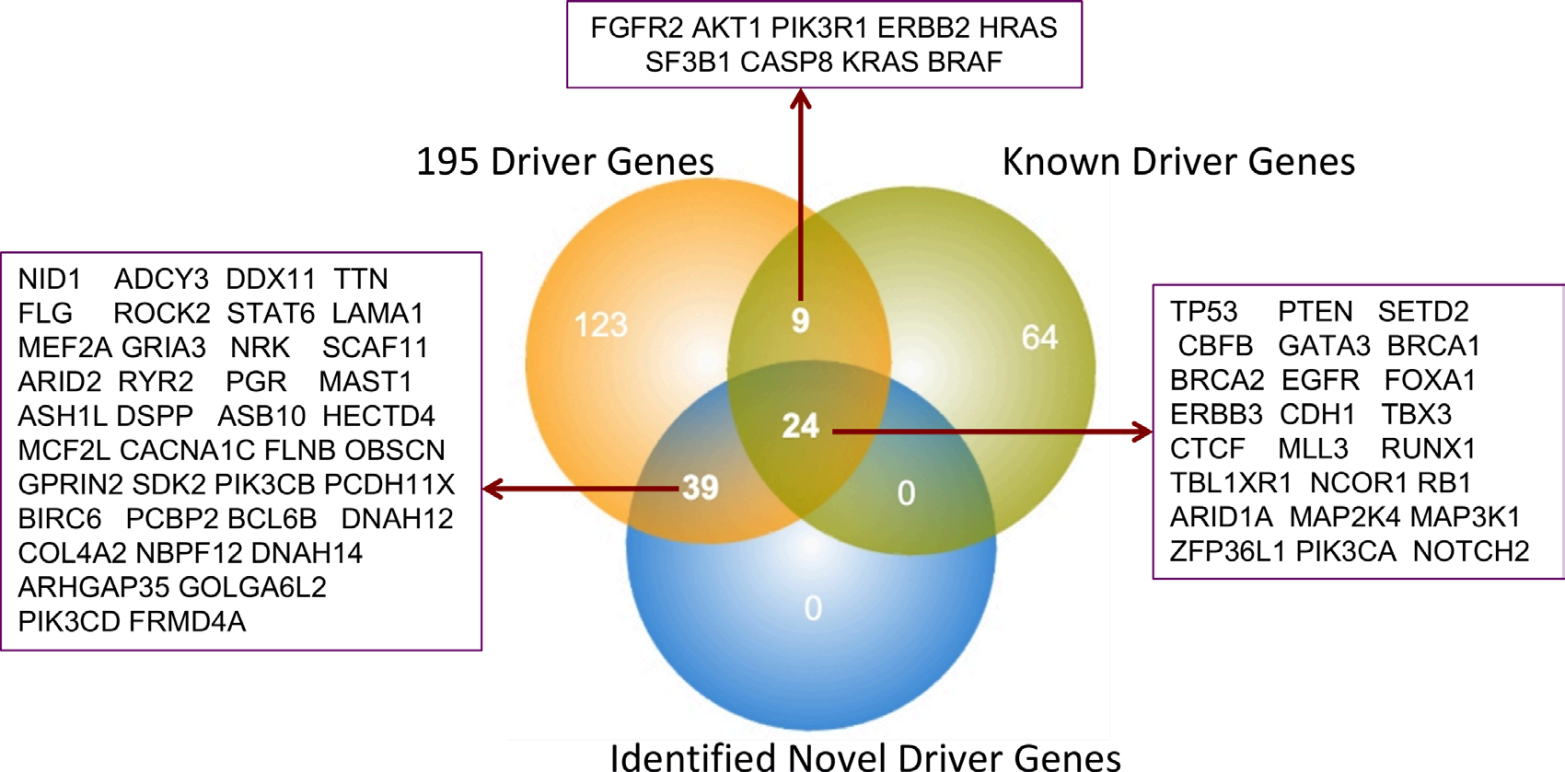
Overall comparisons between published and identified BRCA driver genes

In this study, using a combination of various methodologies, we have analyzed overall 41,948 significant mutations: including 26,448 missense mutations, 1,935 frameshift mutations (InDels), 832 in splice site mutations, as well as 115 and 563 in-frame insertion and in-frame deletion mutations, respectively. As a result of these analyses, we have top listed 63 driver genes, which have a strong correlation with breast cancer subtypes: Luminal A (28.06%), Luminal B (22.01%), basal (19.86%), Her2 (15.82%) and normal (14.23%) breast cancer types. Genes with functionally damaging mutations come after their worst impacts are taken as top candidate (63genes) driver genes. Our data indicate that 24 genes overlap with previously published well-known breast cancer driver genes, whereas the remaining 39 genes that are either not previously highlighted or reported as potential breast cancer drivers (Figure 5). Although recent studies on driver gene identification have developed a vast array of algorithms and resources, yet individual groups follow their own protocols with specific limitations.

Hence, identifying the most potential driver genes are still challenging and also requires the integration of all the results from various tools for comprehensive evaluation. Most of the prior studies on driver gene identification mainly focused to find the driver gene by integrating several computational approaches for filtering out driver genes and their pathways related information relevant to breast cancer. Nevertheless, they failed to validate the identified driver genes with mutation analysis and their impact at transcriptome level. In addition, use of breast cancer patient mutation, mRNAseq expression, and methylation data for the further validation is also lacking in previous studies. In order to provide comprehensive information on breast cancer driver genes we used TCGA-Pan-Cancer breast cancer normal and patient clinical samples, COSMIC mutation data, and methylation as well mRNAseq expression data in the combination with other methodologies (Table 1). This comprehensive information helped us to avoid false positive genes come up during analysis.

For some well-known genes, many functional studies have been carried out. For example, others and we have extensively performed functional studies for p53 and BRCA1 [30, 163–166]. However the final evidence for the majority of other genes, especially for the 39 genes that have not been heighted before as cancer driver requires functional study at various levels, both *in vitro* and *in vivo*, such as gene knockout, knockin, gene overexpression, protein-protein interaction, protein modification, activation and inactivation, and etc. The validation of the mutations affecting regulatory network can be especially changeling. In this case, gene knockout or overexpression may be followed by RNA-sequencing, proteomics and/or epigenetic modifications to uncover alternations of downstream signaling pathways. These studies are vital to perceive the underlying mechanism related to functions of these genes and they will also allow researchers to better understand the tumor heterogeneity, cancer signaling pathway, genetic and epigenetic modifications.

In addition, all the data we have discussed are obtained from sequencing DNA isolated from bulk of each cancer. It is known that genetic instability within individual cancer could generate intratumoral heterogeneity, and that epigenetic modifications may further increase the heterogeneity. These events could significantly affect many aspects of tumorigenesis, including clonal expansion, metastasis, recurrence, drug resistance, and switch off cancer driver during the course of cancer progression. Thus, the use of bulk DNA for sequencing could certainly overshadow the intratumoral heterogeneity. This weakness can be overcome by sequencing DNA isolated from single cancer cells as illustrated by some recent studies [167–170]. Our future efforts will be delivered to analyze the data obtained from the sequencing of single cells, hence, facilitating the discovery of additional therapeutic druggable targets at single cell level for cancer therapies at a personalized fashion.

## SUPPLEMENTARY FIGURES AND TABLES






