# Supplementary material for: Characterization of potential driver mutations involved in human breast cancer by computational approaches
Source: Oncotarget. 2017 Apr 19;8(30):50252–72. doi: 10.18632/oncotarget.17225 (PMC5564847; doi:10.18632/oncotarget.17225)
Supplement: Supplementary file 1 [file oncotarget-08-50252-s001.pdf]

Characterization of potential driver mutations involved in human breast cancer by computational approaches

SUPPLEMENTARY FIGURE AND TABLES

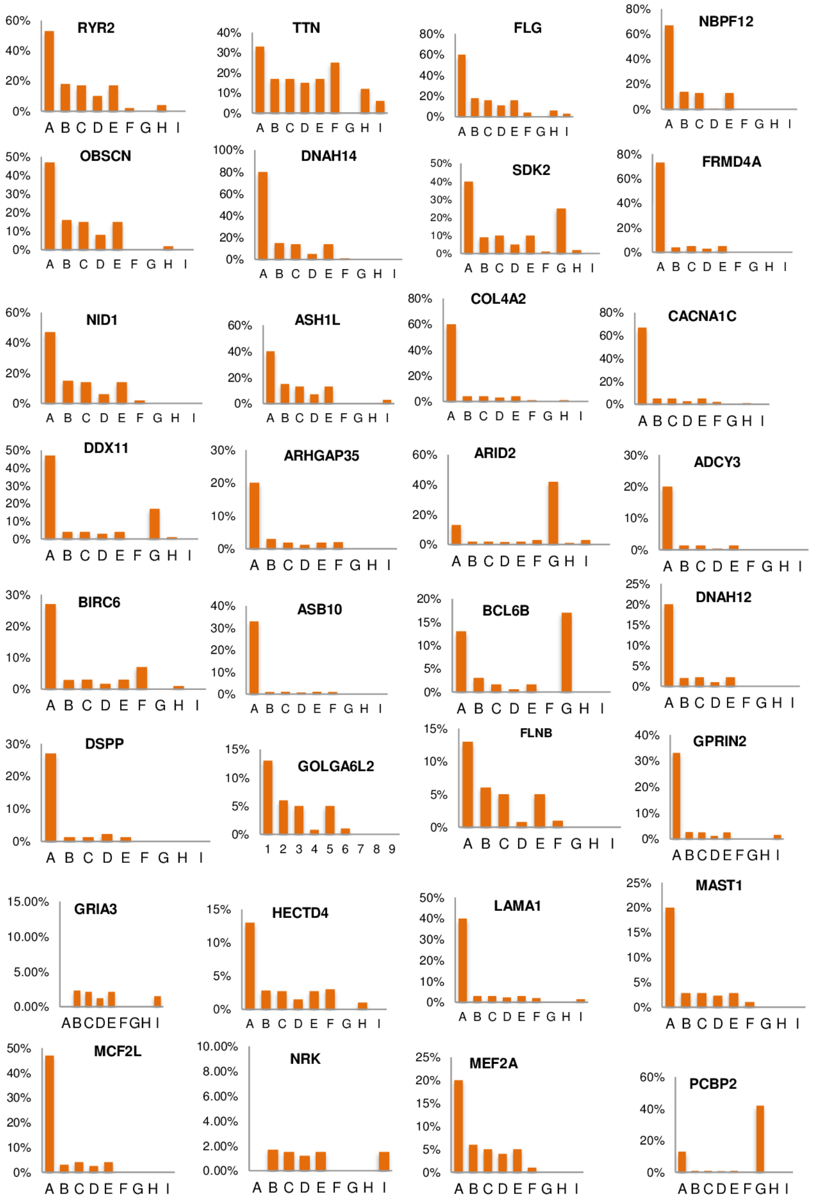

Supplementary Figure 1: Identified individual driver gene mutation percentage over 9 breast cancer data projects (total samples=4621) obtained from cBioportal. (Identified individual driver gene mutation percentage over 9 breast cancer data projects (total samples=4621) obtained from cBioportal. A-Breast cancer patient Xenografts (29samples) (British Columbia, Nature 2014); B-Breast Invasive Carcinoma (816 samples) (TCGA, Cell 2015); C-Breast Cancer (2051 samples) (METABRIC, Nature 2012 & Nat Communications 2016); D-Breast Invasive Carcinoma (482 samples) (TCGA, Nature 2012); E-Breast Invasive Carcinoma (963 samples) (TCGA, Provisional);F-Breast Invasive Carcinoma (100 samples) (Sanger, Nature 2012); G-Adenoid Cystic Carcinoma of the Breast (12samples) (MSKCC, J Pathol. 2015); H-Breast Invasive Carcinoma (103samples) (Broad, Nature 2012);I-Breast Invasive Carcinoma (65) (British Columbia, Nature 2012). No gene mutations in specific projects are represented with blank space).

Supplementary Table 1: Identified 956 Breast Cancer Driver Genes

See Supplementary File: 1

Supplementary Table 2: Identified Breast Cancer Driver Mutation Genes and their mutations

See Supplementary File: 2
