# Supplementary material for: Characterization of potential driver mutations involved in human breast cancer by computational approaches
Source: Oncotarget. 2017 Apr 19;8(30):50252–72. doi: 10.18632/oncotarget.17225 (PMC5564847; doi:10.18632/oncotarget.17225)
Supplement: Supplementary file 2 [file oncotarget-08-50252-s002.docx]

**Supplementary Table 1**

| **Identified Breast Cancer Driver Genes** | | | | | |
| --- | --- | --- | --- | --- | --- |
| AARS | AOAH | BRCA2 | CDH23 | CUBN | ELAC2 |
| ABCA12 | AP1S1 | BRPF3 | CDH24 | CX3CR1 | ELFN2 |
| ABCA2 | APC | BTNL8 | CDK12 | CXXC11 | EML4 |
| ABCA6 | APOBR | BZRAP1 | CDKN1B | CYP11B1 | ENPP2 |
| ABCA7 | AQP7 | C12orf42 | CECR1 | DAAM2 | EP300 |
| ABCA9 | AR | C12orf68 | CECR2 | DAB2 | EPDR1 |
| ABCB10 | ARAF | C14orf180 | CELA1 | DAPK1 | EPPK1 |
| ABCB6 | ARFGEF1 | C15orf59 | CELSR2 | DAZAP1 | ERBB2 |
| ABCC2 | ARHGAP35 | C1QTNF5 | CEP164 | DBH | ERBB3 |
| ABCF1 | ARHGAP5 | C20orf112 | CFP | DCHS1 | ERBB4 |
| ABHD17A | ARHGEF15 | C20orf85 | CHD1L | DCP1B | ERCC6L |
| ABL1 | ARHGEF17 | C2CD5 | CHD3 | DDHD1 | ERLIN2 |
| ABR | ARHGEF38 | C2orf16 | CHD6 | DDX11 | ETV4 |
| ACACA | ARID1A | C7 | CHD8 | DDX27 | EXOC6 |
| ACO1 | ARID2 | C9orf43 | CHERP | DENND4B | EXT1 |
| ACO2 | ARSB | CACNA1A | CHM | DHX32 | F5 |
| ACOT4 | ART5 | CACNA1B | CHRM2 | DHX37 | F8 |
| ACPP | ASB10 | CACNA1C | CHRNB4 | DIAPH1 | FAM104A |
| ACTB | ASCC3 | CACNA1E | CIC | DISP1 | FAM111B |
| ACTG1 | ASF1A | CACNA1F | CLSTN2 | DKC1 | FAM117B |
| ACTL6B | ASH1L | CACNA1S | CLTC | DMD | FAM13C |
| ACTN2 | ASXL2 | CADM1 | CNGA3 | DMKN | FAM171B |
| ADAM23 | ATF6 | CADM2 | CNN2 | DMXL1 | FAM174B |
| ADAMTS1 | ATM | CAMSAP3 | CNOT3 | DMXL2 | FAM21A |
| ADAMTS2 | ATN1 | CAMTA2 | CNPY3 | DNAH12 | FAM21C |
| ADAMTS7 | ATP6V1B1 | CAP1 | COBLL1 | DNAH14 | FAM65A |
| ADAMTSL1 | ATP8B2 | CAPN1 | COL12A1 | DNAH2 | FAM71E2 |
| ADCY3 | ATXN1 | CAPN2 | COL14A1 | DNAH3 | FAM98C |
| ADCY9 | AXIN1 | CASP8 | COL1A2 | DNAJC11 | FANCD2 |
| ADD1 | AXL | CASQ2 | COL4A1 | DNAJC13 | FASN |
| ADRBK1 | B4GALNT1 | CASR | COL4A2 | DND1 | FASTKD3 |
| AHDC1 | BACH2 | CASZ1 | COL4A5 | DNMT1 | FAT1 |
| AIM1 | BAI1 | CBFB | COL5A1 | DSCAM | FAT2 |
| AKAP11 | BAIAP2L2 | CBLB | COL7A1 | DSG3 | FAT3 |
| AKAP4 | BAP1 | CCDC14 | COMP | DSP | FAT4 |
| AKT1 | BAX | CCDC144NL | CPS1 | DSPP | FBN1 |
| AKT2 | BAZ2B | CCDC27 | CRAT | DST | FBN2 |
| AKT3 | BCAR1 | CCDC66 | CREBBP | DUSP10 | FBN3 |
| ALDH2 | BCAR3 | CCND3 | CRIPAK | DYNC2H1 | FBXL17 |
| ALMS1 | BCL11B | CCS | CRISPLD1 | DYSF | FBXO18 |
| ALPP | BCL6B | CD180 | CRMP1 | E2F4 | FBXW7 |
| ALPPL2 | BCL9L | CD1E | CROCC | ECM2 | FBXW9 |
| AMMECR1 | BCR | CD300LG | CRYGA | EEF2K | FCGBP |
| AMPH | BDP1 | CD97 | CSPG4 | EFNB3 | FGFR2 |
| ANGPTL6 | BIK | CDC27 | CT47B1 | EFTUD2 | FGFR3 |
| ANK2 | BIRC6 | CDC42BPG | CTCF | EGF | FGFR4 |
| ANKRD30B | BMP1 | CDC42EP1 | CTNNAL1 | EGFR | FLG |
| ANKRD36C | BPIFB4 | CDC6 | CTNND2 | EGR1 | FLNA |
| ANLN | BRAF | CDH1 | CTSA | EHBP1L1 | FLNB |
| ANO4 | BRCA1 | CDH10 | CTU2 | EIF4G3 | FLNC |
| FLT4 | GRIN2A | INO80 | KRTAP4-5 | MCC | MYO9B |
| FMN2 | GRIN2B | INPP5A | KRTAP4-7 | MCF2L | MYT1 |
| FMNL2 | GRM6 | INPPL1 | KRTAP4-8 | MCTP2 | NACAD |
| FN1 | GTSE1 | IQCC | KRTAP5-1 | MDN1 | NAMPTL |
| FN3K | GUCY2C | IQSEC2 | KRTAP5-5 | MED14 | NAP1L1 |
| FNDC1 | GYLTL1B | IQUB | KRTAP9-1 | MED15 | NASP |
| FNDC4 | HCFC1 | IRF3 | KRTAP9-9 | MEF2A | NBAS |
| FOXA1 | HDAC2 | IRF5 | KSR1 | MEGF8 | NBPF10 |
| FOXJ3 | HDAC6 | IRS1 | LAMA1 | MEIS1 | NBPF12 |
| FOXP4 | HDGFRP2 | IRS4 | LAMA2 | MET | NBPF9 |
| FRAS1 | HECTD4 | ITGAM | LAMA5 | MGA | NCAN |
| FRG1 | HECW1 | ITGB1 | LAMB4 | MGAM | NCAPD3 |
| FRG1B | HELZ2 | ITGB2 | LAMC1 | MICAL3 | NCF4 |
| FRMD4A | HERC1 | ITGB4 | LCA5L | MID1 | NCOA3 |
| FSIP2 | HERC2 | ITGB6 | LCMT2 | MLL2 | NCOR1 |
| FUT2 | HIST1H3B | ITPKB | LCT | MLL3 | NCOR2 |
| FXR1 | HLA-A | ITPR1 | LDLR | MLLT10 | NDUFV3 |
| GABRA4 | HLA-B | ITPR2 | LENG9 | MMEL1 | NEB |
| GABRD | HLA-DRB1 | JPH4 | LGALS9B | MOCOS | NEK1 |
| GAGE2D | HLA-DRB5 | JTB | LIFR | MOV10L1 | NF1 |
| GALNTL6 | HLF | JUN | LIMK1 | MPDZ | NFE2L3 |
| GATA3 | HMCN1 | KBTBD6 | LIMK2 | MPEG1 | NFKB1 |
| GCN1L1 | HMGXB4 | KCNB2 | LIPN | MRPL24 | NFKB2 |
| GDI1 | HNF1A | KCNH1 | LOR | MSH2 | NHS |
| GEMIN4 | HNRNPD | KCNJ12 | LOXL3 | MSH3 | NID1 |
| GIGYF1 | HOXA1 | KCNJ15 | LRBA | MSN | NKTR |
| GIGYF2 | HOXB1 | KCNN2 | LRCH3 | MST1 | NLRC4 |
| GIMAP7 | HOXB3 | KCNN3 | LRP1 | MTBP | NLRP13 |
| GIPC3 | HOXD11 | KCTD19 | LRP1B | MTDH | NMT2 |
| GLG1 | HOXD9 | KDELR1 | LRP2 | MTOR | NOTCH1 |
| GLTSCR1 | HRAS | KEAP1 | LRP4 | MTUS2 | NOTCH2 |
| GLYR1 | HRCT1 | KIAA0226 | LRRK2 | MUC12 | NOTCH3 |
| GNAZ | HRNR | KIAA0922 | LSR | MUC2 | NOTCH4 |
| GNRH2 | HS3ST4 | KIAA0947 | LTBP2 | MUC4 | NPEPPS |
| GOLGA2 | HS6ST1 | KIAA1211 | LURAP1L | MUC5B | NR1H2 |
| GOLGA3 | HS6ST2 | KIAA1239 | MAB21L1 | MUC6 | NR4A2 |
| GOLGA6L2 | HSPA9 | KIAA1462 | MACF1 | MYB | NRCAM |
| GOLGA6L6 | HSPD1 | KIAA1551 | MAFA | MYBL1 | NRK |
| GPR111 | HSPG2 | KIAA2018 | MAGEA8 | MYBPC3 | NUPL2 |
| GPR158 | HUWE1 | KIF14 | MAMSTR | MYCBP2 | OBP2B |
| GPR32 | IDH1 | KIF16B | MAP1A | MYH14 | OBSCN |
| GPR98 | IFI27 | KIF1B | MAP1B | MYH6 | OGFR |
| GPRIN1 | IGDCC4 | KIF4A | MAP2K1 | MYH7B | OLFML2B |
| GPRIN2 | IGSF1 | KRAS | MAP2K4 | MYH9 | ONECUT2 |
| GPS2 | IGSF3 | KRT1 | MAP3K1 | MYL1 | OR10R2 |
| GRB7 | IKBKB | KRT38 | MAP3K10 | MYLK | OR11H1 |
| GRIA1 | IL1RAPL1 | KRT78 | MAP3K4 | MYO18B | OR2T2 |
| GRIA3 | IL27RA | KRT79 | MAPRE3 | MYO5A | OR2T35 |
| GRID1 | IL6 | KRTAP10-7 | MARK2 | MYO7A | OR4D10 |
| GRIK2 | INHBA | KRTAP17-1 | MAST1 | MYO7B | OR5P2 |
| OR6A2 | PIK3CA | PTHLH | RYR3 | SMTN | THNSL2 |
| OR6C76 | PIK3CB | PTPRT | SAAL1 | SMURF2 | THOC1 |
| OR7C1 | PIK3CD | PVRL1 | SAFB2 | SNAPC4 | TICAM1 |
| OTOF | PIK3R1 | PXDN | SARDH | SOS1 | TJP3 |
| OTOP1 | PIK3R3 | PXDNL | SATB2 | SOS2 | TLN1 |
| P2RX2 | PIKFYVE | RAB40C | SBF2 | SPEN | TLR4 |
| PABPC3 | PKD1 | RAB42 | SBNO2 | SPHKAP | TMBIM4 |
| PACS2 | PKHD1L1 | RAF1 | SCAF1 | SPRED3 | TMED10 |
| PAPD7 | PKP1 | RAI1 | SCAF11 | SPTA1 | TMEM184A |
| PAPPA | PLA2G15 | RALGAPA2 | SCML1 | SPTAN1 | TMEM247 |
| PARD3 | PLA2G4A | RALY | SCN10A | SPTB | TMEM48 |
| PARP4 | PLCB4 | RANBP2 | SCN2A | SPTBN2 | TMEM74 |
| PASK | PLCE1 | RARA | SCN3A | SPTBN5 | TMPRSS13 |
| PAX2 | PLCG1 | RARB | SCN4A | SRCAP | TMTC2 |
| PAX6 | PLCG2 | RASGRF2 | SCN9A | SRFBP1 | TNKS |
| PCBP2 | PLK1 | RB1 | SDK2 | SRRM2 | TNPO3 |
| PCDH10 | PLOD3 | RBM12 | SEC14L5 | SRRT | TNRC18 |
| PCDH11X | PLS3 | RBM15B | SELP | SSC5D | TNS1 |
| PCDH12 | PLXNB2 | RBM19 | SELPLG | STAB1 | TOPBP1 |
| PCDHA1 | PMEPA1 | RBM5 | SENP1 | STAT6 | TP53 |
| PCDHA3 | POLA1 | RBMX | SEPTIN10 | STK11 | TP53BP1 |
| PCDHAC2 | POLD1 | RCC1 | SETD2 | SUPT5H | TPD52L2 |
| PCDHB10 | POLE | RELN | SETDB1 | SUPT6H | TPP1 |
| PCDHB11 | POLI | RETSAT | SF3B1 | SV2A | TPP2 |
| PCDHB14 | POLR1A | REV3L | SF3B4 | SVEP1 | TPRX1 |
| PCDHGA1 | POLR2J | RFC4 | SH3PXD2A | SVIL | TPTE2 |
| PCDHGA4 | POM121 | RGL2 | SHROOM4 | SYNE1 | TRIM51 |
| PCDHGB1 | POMT2 | RGPD3 | SIGLEC1 | SYNE2 | TRIM6-TRIM34 |
| PCED1A | POTEE | RHO | SIGLEC7 | TAF1B | TRIOBP |
| PCK2 | POU4F1 | RHOA | SIK3 | TAF1L | TRPM5 |
| PCNT | POU4F2 | RHPN2 | SKIDA1 | TAS2R43 | TRPM6 |
| PCSK9 | PPP1R12B | RNF113A | SLC10A4 | TBC1D4 | TRPV2 |
| PDCD11 | PPP1R3B | RNF17 | SLC16A2 | TBC1D8 | TSC22D1 |
| PDE1C | PPP4R4 | RNF213 | SLC23A2 | TBL1XR1 | TSHR |
| PDE3A | PRKAR1A | RNPC3 | SLC25A5 | TBX18 | TSPEAR |
| PDE4B | PRKCA | ROCK1 | SLC26A9 | TBX22 | TTN |
| PDGFRB | PRKCB | ROCK2 | SLC30A5 | TBX3 | TYRP1 |
| PEAR1 | PRKDC | ROR2 | SLC38A10 | TCERG1 | U2AF2 |
| PER3 | PRKG2 | RP11-419C5.2 | SLC4A11 | TCF7L2 | UBR5 |
| PEX5L | PRKRA | RP1L1 | SLC4A4 | TCOF1 | UBXN11 |
| PFKP | PRMT8 | RPGR | SLC4A5 | TCP11 | UNC13A |
| PGR | PRPF8 | RPL22 | SLC9A2 | TEAD2 | UNC79 |
| PHC2 | PRRG3 | RPS6KA1 | SLITRK2 | TEP1 | USH2A |
| PHC3 | PRRX1 | RREB1 | SLK | TERF2IP | USP32 |
| PHF12 | PRSS53 | RSPH6A | SMAD2 | TET3 | USP34 |
| PHF2 | PTCD3 | RTEL1 | SMAD4 | TEX15 | USP36 |
| PHLDA1 | PTCH2 | RUNX1 | SMARCA4 | TFAM | USP8 |
| PHLPP1 | PTCHD1 | RUNX1T1 | SMARCAL1 | TFE3 | UTP3 |
| PHOSPHO1 | PTEN | RYR1 | SMARCC2 | TG | UXS1 |
| PIGO | PTH2 | RYR2 | SMG1 | THEM5 | VCPIP1 |
| VEZF1 | WNK1 | ZBTB4 | ZMYND8 | ZNF717 | ZNF831 |
| VILL | WNT9A | ZBTB41 | ZNF185 | ZNF75D | ZNF841 |
| VIT | WRN | ZC3H18 | ZNF302 | ZNF772 | ZSWIM8 |
| VPS13A | WTAP | ZEB2 | ZNF362 | WDFY4 | XRN1 |
| VPS13B | WWC3 | ZFHX3 | ZNF384 | WDR44 | ZAN |
| VPS13D | XDH | ZFP36L1 | ZNF467 | WDR55 | ZIC2 |
| VPS18 | XPC | ZFP36L2 | ZNF585A | ZMYM3 | ZIC3 |
| VWF | XPO1 | ZFP64 | ZNF592 | WDR73 | WDTC1 |
| WBP1 | XPO5 | ZHX1 | ZNF687 | WDR81 |  |
