# Supplementary material for: Characterization of potential driver mutations involved in human breast cancer by computational approaches
Source: Oncotarget. 2017 Apr 19;8(30):50252–72. doi: 10.18632/oncotarget.17225 (PMC5564847; doi:10.18632/oncotarget.17225)
Supplement: Supplementary file 3 [file oncotarget-08-50252-s003.docx]

**Supplementary Table 2**

| **Gene** | **Chromosome** | **Type of Mutation** | **Nucleotide** | **Amino acids** | **BRCA subtypes** |
| --- | --- | --- | --- | --- | --- |
| ABCA2 | chr9:g139911974C>T | Missense | 2382G>A;2379G>A | A794A; A793A | D&L |
| ABCB10 | chr1:g.229683295G>C | Stop Gained | 872C>G | S291* | D&L |
| ACTN2 | chr1:g.236925894C>A | Missense | 1142C>A;2660C>A | A381E;A887E | D&L |
| ADCY3 | chr2:g.25044484C>G | Missense | 1970G>C;1790G>C;3029G>C | W657S;W597S;W1010S | D&L |
| AHDC1 | chr1:g.27874450->G | Frameshift | - | G1393A? | D&L |
| AKAP11 | chr13:g.42877453C>T | Missense | 4571C>T | T1524I | ER+,HER2-VE |
| AKT1 | chr15:g.34147065C>T | Missense | 49C>A;235C>A | Q17K;Q79K | TNBC/LOBU/OTHER;ER+VE,HER-VE |
| AKT3 | chr1:g.243716076G>C | Missense | 1118C>G | A373G | D&L |
| ANK2 | chr4:g.114278161C>T | Missense | 8387C>T;8288C>T | P2796L;P2763L | ER+,HER2-VE |
| AOAH | chr7:g.36671662C>G | Missense | 405G>C;501G>C | K135N;K167N | ER+,HER2-VE |
| ARHGAP35 | chr19:g.47492898G>A | Missense | 4002G>A | - | D&L |
| ARID1A | chr1:g.27097631C>T | Missense | 3220C>T;2071C>T | R1074W; R691W | ER+,HER2-VE |
| ARID2 | chr12:g.46211620G>T | Missense | 586G>T;139G>T | D196Y;D47Y | ER+,HER2-VE |
| ART5 | chr11:g.3661293C>T | Missense | 234G>A;366G>A | E78E;E122E | D&L |
| ASB10 | chr7:g.140439727C>T | Missense | 420G>C;285G>C | W140C;W95C | TNBC/LOBU/OTHER |
| ASH1L | chr1:g.155311856G>A | Missense | 8331C>T;8346C>T | I2777I;I2782I | D&L |
| ATN1 | chr12:g.7043656C>T | Missense | 194C>T | P65L | D&L |
| B4GALNT1 | chr12:g.58021921C>T | Missense | 962G>A | R321Q | ER+,HER2-VE |
| BCL6B | chr17:g.6930108G>T | Missense | 206G>T;1139G>T | G69V;G380V | D&L |
| BCL9L | chr11:g.118779095G>A | Missense | 296C>T | A99V | D&L |
| BCR | chr22:g.23656229A>C | Missense | 3400A>C; 3532A>C | T1134P; T1178P | D&L |
| BIRC6 | chr2:g.32770872C>T | Missense | 12755C>T | S4252F | D&L |
| BRAF | chr16:g.68849565G>A | Missense | 833G>A;2012G>A | R278Q;R671Q | TNBC/LOBU/OTHER |
| BRCA1 | chr17:g.41258536T>G | Missense | 8A>C;149A>C | L3T;K50T | D&L |
| BRCA2 | chr14:g.105243048G>T | Missense | 7948G>C | E2650Q | D&L |
| BRPF3 | chr6:g.36168630T>A | Missense | 531T>A | S177R | ER+,HER2-VE |
| BTNL8 | chr5:g.180374624G>T | Missense | 165G>T;786G>T | Q55H;Q262H;Q137H;Q146H;Q78H | D&L |
| C12ORF68 | chr12:g.48578409->G | Frameshift | - | L168L? | D&L |
| C1QTNF5 | chr11:g.119210189->C | Frameshift | - | A199A? | D&L |
| C9ORF43 | chr9:g.116191234A>C | Missense | 1162A>C | S388R | D&L |
| CACNA1A | chr13:g.32936802G>C | Missense | - | - |  |
| CACNA1B | chr9:g.140880937C>G | Missense | 1842C>G;1845C>G | F614L;F615L | D&L |
| CACNA1C | chr12:g.2705125C>A | Missense | 2824C>A;2749C>A | L942M;L917M | ER+,HER2-VE |
| CACNA1E | chr3:g.178936093G>C | Missense | 601G>T;748G>T | A201S;A250S | TNBC/LOBU/OTHER |
| CASP8 | chr2:g.202131411C>T | Stop Gained | 379C>T; 202C>T | R127*;R68* | D&L |
| CBFB | chr7:g.150883933C>G | Stop Gained | 154G>T;454G>T | E52*;E152* | TNBC/LOBU/OTHER |
| CDC27 | chr17:g.45234484T>C | Missense | 637A>G;545A>G | N213D;N152D | D&L |
| CDC42BPG | chr11:g.64600123C>G | Missense | 2958G>C | L986L | D&L |
| CDH1 | chr13:g.25073471A>G | Missense | 1285G>A;1468G>A | E429K; E490K | TNBC/LOBU/OTHER |
| CELA1 | chr12:g.51737591C>G | Missense | 146G>C | G49A | D&L |
| CLTC | chr17:g.57761111C>T | Missense | 4317C>T;1128C>T | F1439F; F376F | ER+,HER2-VE |
| COL4A2 | chr13:g.111088619G>A | Missense | 730G>A | D244N | D&L |
| CRIPAK | chr4:g.1389289C>T | Missense | 990C>T | P330P | D&L |
| CTCF | chr16:g.67655431G>T | Stop Gained | 310G>T;1294G>T | E104*; E432* | D&L |
| DCHS1 | chr11:g.6661088G>A | Missense | 1757C>T | S586L | ER+,HER2-VE |
| DDX11 | chr12:g.31247702G>A | Missense | 1428G>A; 1350G>A | K476K;K450K | D&L |
| DENND4B | chr1:g.153916630C>- | Frameshift | G85;G74 | - | D&L |
| DNAH12 | chr3:g.57443481C>G | Missense | 3303G>C;3234G>C | R1101R; R1078R | D&L |
| DNAH14 | chr1:g.225230754A>- | Frameshift |  | E569;E588 | D&L |
| DSPP | chr4:g.88535003G>T | Stop Gained | 1189G>T | E397* | D&L |
| EGFR | chr7:g.55211097G>A | Missense | 181G>A;340G>A | E61K;E114K | D&L |
| EP300 | chr22:g.41521913C>T | Stop Gained | 775C>T | Q259* | D&L |
| ERBB2 | chr17:g.37881003->GGCTCCCCA | Inframe Insertion | - | G7448GSPG;G502GSPG;G763GSPG;G778GSPG; G146GSPG | ER+,HER2-VE |
| ERBB3 | chr12:g.56482607C>T | Missense | 887C>T; 1064C>T | T296I;T355I | D&L |
| FAM13C | chr10:g.61083827C>T | Missense | 115G>A;364G>A | V39M;V122M | D&L |
| FAM21A | chr10:g.51859751C>A | Missense | 1298C>A;1562C>A | S433Y;S521Y | D&L |
| FAM21C | chr1:g.181548339G>T | Missense | 1252C>G;1348C>G;1420C>G | R418G;R450G;R474G | TNBC/LOBU/OTHER |
| FBN1 | chr15:g.48787352G>A | Missense | 2645C>T | A882V | D&L |
| FBXO18 | chr10:g.5948251A>C | Missense | 409A>C;562A>C | T137P;T188P | D&L |
| FGFR2 | chr1:g.145299908C>T | Missense | 918C>G;1185C>G;921C>G;501C>G;840C>G;1188C>G;849C>G;843C>G | V306V;V395V;V307V;V167V;V280V;V396V;V283V;V281V | TNBC/LOBU/OTHER |
| FLG | chr1:g.152285314C>T | Missense | 2048G>A | R683H | ER+,HER2-VE |
| FLNA | chrX:g.153582335T>A | Missense | 207A>T;5514A>T;5634A>T;480A>T;5610A>T | G69G;G1838G;G1878G;G160G;G1870G | D&L |
| FLNB | chr3:g.58084468T>G | Missense | 671T>G;1178T>G | V224G;V393G | D&L |
| FMN2 | chr1:g.240519171G>A | Missense | 609G>A;4821G>A | Q203Q;Q1607Q | D&L |
| FOXA1 | chr14:g.38061410C>- | Frameshift | - | L160;L193 | D&L |
| FRG1 | chr4:g.190881957G>A | Missense | 208G>A;592G>A | D70N;D198N | ER+,HER2-VE |
| FRG1B | chr20:g.29624046C>T | Missense | 70C>T;85C>T | P24S;P29S | D&L |
| FRMD4A | chr10:g.13699219C>T | Missense | 2325G>A;2370G>A | A775A;A790A | D&L |
| GATA3 | chr10:g.8115922->C | Frameshift | - | H424P?;H423P? | D&L |
| GOLGA6L2 | chr15:g.23686764C>G | Missense | 39G>C;858G>C | K13N; K286N | D&L |
| GPR32 | chr19:g.51274579C>T | Missense | 722C>T | A241V | ER+,HER2-VE |
| GPRIN2 | chr10:g.46999897G>A | Missense | 1017G>A | A339A | ER+,HER2-VE |
| GRIA3 | chrX:g.122528948G>A | Missense | 880G>A;832G>A | E294K;E278K | D&L |
| HECTD4 | chr12:g.112720942G>C | Missense | 318C>G; 1068C>G | I106M;I356M | D&L |
| HLA-DRB1 | chr6:g.32552137G>A | Missense | 119C>T | P40L | D&L |
| HRAS | chr10:g.46250563C>G | Missense | 196G>A | A66T | TNBC/LOBU/OTHER |
| HRNR | chr1:g.152191390G>A | Missense | 2715C>T | S905S | D&L |
| HS6ST1 | chr2:g.129075961T>C | Missense | 177A>G | T59T | D&L |
| HUWE1 | chrX:g.53588774G>A | Missense | 4549C>T;7450C>T | R1517C;R2484C | D&L |
| ITGB4 | chr17:g.73725372->C | Frameshift | - | D60A?; D198A? | D&L |
| ITPKB | chr1:g.226923383G>C | Missense | 1777C>G | L593V | AMP.OF HER2 GENE; ER+VE, HER2-VE |
| ITPR1 | chr3:g.4699856G>T | Stop Gained | 1000G>T; 1045G>T | E334*;E349* | D&L |
| ITPR2 | chr12:g.26750024C>G | Missense | 4046G>C | R1349T | ER+,HER2-VE |
| KCNH1 | chr1:g.210857470C>T | Missense | 2042G>A;2123G>A | R681Q;R708Q | D&L |
| KCNN3 | chr1:g.154794655G>T | Missense | 939C>A;24C>A | S313S;S8S | ER+,HER2-VE |
| KIAA1551 | chr12:g.32138635G>A | Missense | 4746G>A | L1582L | ER+,HER2-VE |
| KRAS | chr12:g.25362806G>A | Stop Gained | 490C>T;151C>T | R164*;R51* | ER+,HER2-VE |
| KRT1 | chr12:g.53072381G>A | Missense | 751C>T | R251W | D&L |
| KRT78 | chr12:g.53233250G>A | Missense | 960C>T;1290C>T | S320S;S430S | D&L |
| KRT79 | chr12:g.53215777->C | Frameshift | - | A496A? | D&L |
| KRTAP5-1 | chr11:g.1606146->GCC | Inframe Insertion | - | C112WR | D&L |
| LAMA1 | chr18:g.7012038G>T | Missense | 3463C>A | L1155M | ER+,HER2-VE |
| LRP1 | chr12:g.57584777C>T | Missense | 276C>T;7221C>T | D92D;D2407D | ER+,HER2-VE |
| LRRK2 | chr12:g.40668472C>T | Missense | 988C>T;1744C>T | L330L;L582L | ER+,HER2-VE |
| MAP2K1 | chr15:g.66774131G>A | Missense | 607G>A;79G>A | E203K;E27K | D&L |
| MAP2K4 | chr17:g.12043179G>C | Missense | 1097G>C;1064G>C | R366T;R355T | ER+,HER2-VE |
| MAP3K1 | chr5:g.56178085C>T | Stop Gained | 3058C>T | Q1020* | D&L |
| MAP3K4 | chr6:g.161470388G>T | Stop Gained | 1084G>T | E362* | D&L |
| MAST1 | chr19:g.12954404C>G | Missense | 169C>G;310C>G;298C>G | P57A;P104A;P100A | ER+,HER2-VE |
| MCF2L | chr13:g.113719306C>T | Missense | 657C>T;834C>T;753C>T;423C>T;681C>T;762C>T;663C>T;675C>T | F219F;F278F;F251F;F141F;F227F;F254F;F254F;F221F;F225F | D&L |
| MEF2A | chr15:g.100211585G>C | Missense | 112G>C;316G>C | E38Q;E106Q | D&L |
| MGAM | chr7:g.141805647A>C | Missense | 5530A>C;8218A>C | S1844R;S2740R | ER+,HER2-VE |
| MLL2 (KMT2D) | chr12:g.49416396G>C | Missense | 16315C>G;358C>G | R5439G;R120G | ER+,HER2-VE |
| MLL3(KMT2C) | chr7:g.151860831T>G | Missense | 2346A>C;9831A>C | P782P;93277P | D&L |
| MMEL1 | chr1:g:2560819CAG>- | Inframe Deletion | - | L35;L26 | D&L |
| MRPL24 | chr1:g.156707244C>G | Missense | 597G>C | M199I | D&L |
| MTOR | chr1:g.11177099G>A | Missense | 1593C>T;6978C>T | V531V;V2326V | D&L |
| MTUS2 | chr13:g.30014156A>G | Missense | 91A>G;3184A>G | I31V;I1062V | ER+,HER2-VE |
| MYLK | chr3:g.123444902G>A | Missense | 1540C>T;1333C>T | P514S;P445S | D&L |
| NAP1L1 | ;chr17:g.7577526A>G | Missense | 121C>G;103C>G | Q41E;Q35E | AMP.OF HER2GENE; ER+, HER-VE |
| NBPF10 | chr16:g.67116170G>T | Missense | 144C>T;957C>T | S48S; S319S | TNBC/LOBU/OTHER |
| NBPF12 | chr1:g.146400130C>A | Missense | 381C>A; 606C>A | D127E;D202E | D&L |
| NCOA3 | chr20:g.46264773T>G | Missense | 1643T>G;1673T>G | L548W;L558W | D&L |
| NCOR1 | chr17:g.16068397A>- | Frameshift | - | S172;S63 | D&L |
| NCOR2 | chr12:g.124816871C>A | Stop Gained | 19G>T;511G>T;5581G>T;268G>T;6898G>T;6919G>T;6871G>T;6868G>T | E7*;E171*;E1861*;E90*;E2307*;E2291*;E2290* | D&L |
| NID1 | chr1:g.236143802C>T | Missense | 2980G>A;3379G>A | D994N;D1127N | D&L |
| NOTCH2 | chr1:g.120539920G>A | Missense | 334C>T;451C>T | H112Y;H151Y | D&L |
| NR1H2 | chr19:g.50881919G>A | Missense | 322G>A;613G>A | E108K;E205K | ER+,HER2-VE |
| NRK | chrX:g.105153210T>C | Missense | 1580T>C;1577T>C | V527A;V526A | ER+,HER2-VE |
| OBSCN | chr1:g.228430973G>T | Missense | 3295G>T;3019G>T | A1099S;A1007S | ER+,HER2-VE |
| OGFR | chr20:g.61444665G>A | Missense | 1698G>A;1542G>A | P566P;P514P | D&L |
| OLFML2B | chr1:g.161967735C>T | Missense | 1354G>A;1357G>A | V452M;V453M | D&L |
| OR2T2 | chr1:g.248616513C>T | Missense | 415C>T | R139C | D&L |
| OR2T35 | chr1:g.248801953->T | Frameshift | - | C203*? | D&L |
| OR5P2 | chr11:g.7818269G>A | Missense | 221C>T | T74I | D&L |
| OR6C76 | chr12:g.55820676C>G | Missense | 639C>G | L213L | D&L |
| OTOF | chr2:g.26693990A>C | Missense | 1823T>G;1592T>G;3893T>G | V608G;V531G;V1298G | D&L |
| PABPC3 | chr13:g.25671591C>G | Missense | 1255C>G | P419A | D&L |
| PARD3 | chr10:g.34400148C>T | Missense | 3909G>A;3840G>A;3750G>A;4011G>A;3882G>A;4020G>A;3684G>A;3972G>A | A1303A;A1280A;A1250A;A1337A;A1294A;A1340A;A1228A;A1324A | ER+,HER2-VE |
| PARP4 | chr12:g.3649862C>T | Missense | 445T>C | F149L | TNBC/LOBU/OTHER |
| PCBP2 | chr12:g.53856277->C | Frameshift | - | S44L?;S169L?;S153L?;S3L?;S173L? | D&L |
| PCDH11X | chrX:g.91873468G>T | Missense | 3519G>T;3462G>T;3543G>T;3573G>T;3549G>T | Q1173H;Q1154H;Q1181H;Q1191H;Q1183H | D&L |
| PCDHA3 | chr5:g.140180891G>A | Missense | 109G>A | E37K | D&L |
| PCDHGA4 | chr5:g.140735310C>T | Missense | 543C>T | D181D | D&L |
| PCDHGB1 | chr12:g.76461236G>C | Missense | 2060A>G | Y687C | AMP.OF HER2 GENE; ER+, HER-VE |
| PDE3A | chr12:g.20787910G>A | Missense | 1921G>A | D641N | D&L |
| PDGFRB | chr5:g.149497273A>G | Missense | 3045T>C | N1015N | ASIAN PHENOTYPE |
| PFKP | chr10:g.3149487G>A | Missense | 832G>A;856G>A208G>A | E278K;E286K;E70K | D&L |
| PGR | chr11:g.100996740->C | Frameshift | - | E2E?; E596E? | D&L |
| PHLDA1 | chr12:g.76424820G>T | Missense | 279C>A;702C>A | F93L;F234L | D&L |
| PIK3CA | chr5:g.140731887A>G | Missense | 1635G>C | E545D | AMP.OF HER2GENE; ER+, HER-VE; TNBC/LOB/OTHERS |
| PIK3CB | chr3:g.138426040C>G | Missense | 1491G>C;435G>C | E497D;E145D | D&L |
| PIK3CD | chr1:g.9784441T>C | Missense | 2898T>C;2826T>C | I966I;I942I | ER+,HER2-VE |
| PIK3R1 | chr5:g.67576546C>G | Missense | 825C>G;15C>G | F275L;F5L | ER+,HER2-VE |
| PLCG2 | chr16:g.81925098C>T | Stop Gained | 889C>T;196C>T | R297*;R66* | ER+,HER2-VE |
| POLE | chr12:g.133219516T>C | Missense | 4618A>G;4537A>G | K1540E;K1513E | D&L |
| PRKCB | chr16:g.24104239C>G | Missense | 102G>C;657C>G | L34L;L219L | D&L |
| PRMT8 | chr11:g.533860C>T | Missense | 139C>T;166C>T | P47S;P56S | TNBC/LOBU/OTHER |
| PTEN | chr10:g.89624285G>C | Missense | 59G>A | G20A | ER+,HER2-VE |
| PTHLH | chr12:g.28116584G>A | Missense | 245C>T;221C>T | A82V;A74V | D&L |
| RAF1 | chr3:g.12647703A>T | Missense | 677T>A;434T>A | V226D;V145D | D&L |
| RB1 | chr13:g.48934189C>A | Stop Gained | 644C>A | S215* | D&L |
| RBM5 | chr3:g.50137998A>G | Missense | 443A>G;351A>G | Y148C;L117L(SYNON) | D&L |
| RBMX | chrX:g.135960220A>C | Missense | 242T>G;245T>G | V81G;V82G | D&L |
| RELN | chr7:g.103113290C>T | Missense | 10346G>A;10352G>A | R3449K;R3451K | D&L |
| RNF17 | chr13:g.25399899C>G | Missense | 2234C>G;206C>G | A745G;A69G | D&L |
| ROCK2 | chr2:g.11334430C>T | Missense | 2831G>A;3560G>A | S944N;S1187N | D&L |
| RPS6KA1 | chr1:g.26882031G>A | Missense | 798G>A;783G>A;858G>A;831G>A;555G>A | A266A;A261A;A286A;A277A;A185A | D&L |
| RUNX1 | chr21:g.36252995C>- | Frameshift | - | D123;D96;D110;D111;D99 | D&L |
| RYR2 | chr1:g.237954734T>C | Missense | 13434T>C;13482T>C13500T>C | Y4478Y;Y4494Y;Y4500Y | D&L |
| RYR3 | chr10:g.123274733G>C | Missense | 13944C>T;13959C>T | F4648F;F4653F | TNBC/LOBU/OTHER |
| SAAL1 | chr11:g.18127519C>G | Missense | 46G>C;70G>C | D16H;D24H | ER+,HER2-VE |
| SCAF11 | chr12:g.46320764G>A | Missense | 2144C>T;1775C>T;2720C>T | S715F;S592F;S907F | D&L |
| SDK2 | chr17:g.71397224G>A | Missense | 434C>T;2906C>T | A145V;A969V | D&L |
| SELPLG | chr12:g.109017672GCA>- | Distruptive Inframe Deletion | - | VP137A;VP153A | D&L |
| SENP1 | chr12:g.48491904T>A | Missense | 104A>T;8A>T | D35V;D3V | ER+,HER2-VE |
| SEPTIN10 | chr2:g.110303647C>T | Missense | 828G>A;930G>A;1260G>A;1392G>A;1284G>A | R276R;R310R;R420R;R443R;R428R | ER+,HER2-VE |
| SETD2 | chr3:g.47163294A>C | Missense | 2700T>G;2832T>G | A900A;A944A | D&L |
| SF3B1 | chr2:g.198267459G>A | Missense | 1898C>T | A633V | D&L |
| SH3PXD2A | chr10:g.105484102G>A | Missense | 186C>T;324C>T | P62P;P108P | ER+,HER2-VE |
| SLC38A10 | chr17:g.79263495G>T | Missense | 69C>A;213C>A | G23G;G71G | ER+,HER2-VE |
| SMARCC2 | chr12:g.56565153A>C | Missense | 2156T>G;2249T>G | V719G;V750G | D&L |
| SOS1 | chr2:g.39281774G>C | Stop Gained | 530C>G;701C>G | S177*;S234* | D&L |
| STAT6 | chr12:g.57500358G>A | Missense | 154C>T;484C>T | L52L;L162L | D&L |
| TBL1XR1 | chr3:g.176769295->TA | Frameshift | - | A55V?;A142V? | D&L |
| TBX3 | chr12:g.115115442T>C | Missense | 884A>G;824A>G | D295G;D275G | ER+,HER2-VE |
| TFAM | chr10:g.60148570A>- | Frameshift | - | T125;T144 | D&L |
| THEM5 | chr1:g.151823599A>G | Missense | 394T>C;40T>C | F132L;F14L | D&L |
| TMBIM4 | chr12:g.66531907C>T | Missense | 19G>A;550G>A;457G>A;691G>A;467G>A | A7T:A184T;A153T;A231T;R156H | D&L |
| TMTC2 | chr12:g.83250926G>A | Missense | 221G>A;203G>A | R74H;R68H | D&L |
| TP53 | chr17:g.7577127C>T | Missense | 811G>A;415G>A359T>C;755T>C | E27K;E139K;L120P;L252P | HER+VE,HER-VE; D&L |
| TP53BP1 | chr15:g.43784254G>A | Stop Gained | 217C>T;232C>T | R73*;R78* | ASIAN PHENOTYPE |
| TPRX1 | chr19:g.48306222G>A | Missense | 46C>T;337C>T | R16C;R113C | D&L |
| TSC22D1 | chr13:g.45010182T>C | Missense | 2962A>G;175A>G | M988V;M59V | D&L |
| TTN | chr2:g.179621018G>C | Missense | 10672C>G;11185C>G | L3558V;L3729V | ER+,HER2-VE |
| UBXN11 | chr1:g.26620739A>T | Missense | 516T>A;417T>A;402T>A;42T>A;291T>A | H172Q;H139Q;H134Q;H14Q;H97Q | D&L |
| USP36 | chr17:g.76803587G>A | Missense | 1539C>T;639C>T | S513S;S213S | ER+,HER2-VE |
| VEZF1 | chr17:g.56051938G>C | Missense | 1435C>G;916C>G;1462C>G | L479V;L306V;L488V | ER+,HER2-VE |
| VWF | chr12:g.6230460G>A | Stop Gained | 100C>T;211C>T | R34*;R71* | D&L |
| WDFY4 | chr10:g.50171988C>A | Missense | 8325C>A;2583C>A | I2775I;I861I | D&L |
| WDTC1 | chr1:g.27632845G>A | Missense | 2005G>A;2002G>A | E669K;E668K | ER+,HER2-VE |
| WNT9A | chr1:g.228109385C>T | Missense | 932G>A | R311H | ER+,HER2-VE |
| ZFP36L1 | chr14:g.69256798->G | Frameshift | - | L163P?;L135P?;L157P? | ER+,HER2-VE |
| ZNF302 | chr19:g.35175898G>C | Missense | 56G>C;1088G>C | G319A;G363A | ER+,HER2-VE |
| ZNF384 | chr12:g.6787583G>A | Missense | 348c>t; 396C>T | T116T; T132T | D&L |

1. D&L-Ductal and Lobular; TNBC-Triple negative breast cancer; LOBU-Lobular carcinoma.

2. Germline mutations in BRCA1 and BRCA2 occur at about 2-5% of different populations of cancer patient. But our analysis indicated that the BRCA1 (Missense 8A>C; 149A>C L3T; K50T) and BRCA2 (7948G>C E2650Q) mutations described here are not germline mutation. We have used the following three approaches for the analysis: 1) we checked datasets of BRCA1 (<https://research.nhgri.nih.gov/projects/bic/circos/>) and BRCA2 (<http://www.cancerindex.org/geneweb.BRCA2.htm>); 2) we analyzed OMIM database (<https://www.omim.org>) using identifier, MIM#113705 (for BRCA1) and MIM#600185 (for BRCA2); and 3) we also searched PubMed for these mutations and could not find any reports about these mutations either. Therefore these mutations are likely somatic mutations.
